# Relaxor behavior in rocksalt cation-ordered material induced by (anti)ferroelectric phase competition

**DOI:** 10.1038/s41467-026-74237-z

**Published:** 2026-06-23

**Authors:** Yu Yun, Liyan Wu, Drew Behrendt, Hao Pan, Menglin Zhu, Michael Xu, Anthony J. Ruffino, John Carroll, Irina Baraban, Zishen Tian, Xianfei Xu, Dongfang Chen, Brendan M. Hanrahan, Lane W. Martin, James M. LeBeau, Andrew M. Rappe, Ilya Grinberg, Jonathan E. Spanier

**Affiliations:** 1 Department of Mechanical Engineering and Mechanics, Drexel University, Philadelphia, PA USA; 2 Department of Chemistry, University of Pennsylvania, Philadelphia, PA USA; 3 Department of Materials Science and Engineering, University of California, Berkeley, CA USA; 4 Department of Materials Science & Engineering, MIT, Cambridge, MA USA; 5 Department of Electrical and Computer Engineering, Drexel University, Philadelphia, PA USA; 6 Department of Physics, Drexel University, Philadelphia, PA USA; 7 DEVCOM, Army Research Laboratory, Adelphi, MD USA; 8 Department of Materials Science and Nanoengineering, Rice University, Houston, TX USA; 9 Department of Chemistry, Rice University, Houston, TX USA; 10 Department of Physics and Astronomy, Rice University, Houston, TX USA; 11 Rice Advanced Materials Institute, Rice University, Houston, TX USA; 12 Department of Chemistry, Bar-Ilan University, Ramat Gan, Israel; 13 Department of Materials Science & Engineering, Drexel University, Philadelphia, PA USA

**Keywords:** Ferroelectrics and multiferroics, Atomistic models, Phase transitions and critical phenomena, Ferroelectrics and multiferroics

## Abstract

Relaxor ferroelectrics are characterized by dispersion of the temperature-dependent dielectric constant with frequency and enhanced electromechanical coupling. These properties arise from the dynamic polar response of correlated nanodomains that are strongly associated with atomic-scale compositional disorder, which disrupts long-range ferroelectric ordering and enables nanodomain formation. Here, we report relaxor properties originating from spontaneous low temperature phase competition in fully cation-ordered antiferroelectric PbMg_0.5_W_0.5_O_3_ epitaxial films, including the identification of a new low-energy polar phase. Unlike prototypical relaxors, the *B*-site cations in coherently strained PbMg_0.5_W_0.5_O_3_ films exhibit long-range rocksalt chemical ordering. Temperature-dependent polarization studies reveal the switching behaviors associated with the phase transitions from paraelectric to antiferroelectric to ferroelectric, and the characteristic dielectric relaxation is ascribed instead to phase competition between the polar and antipolar phases mediated by temperature and substrate clamping. This phase competition breaks long-range dipole correlation and leads to dielectric dispersion and relaxor behavior. These findings demonstrate a new paradigm for designing relaxor material properties through engineered phase competition.

## Abstract (web summary)

The authors show that relaxor behavior can emerge in a fully cation-ordered oxide through competition between ferroelectric and antiferroelectric phases, revealing a new route to designing high-performance materials without chemical disorder.

## Introduction

Relaxor ferroelectrics (FE) are prized for their exceptional electromechanical and dielectric responses, making them vital for sensors, transducers, and energy storage^1–3^. Their unique properties are related to fascinating, complex polar dynamics and a flat Landau energy profile, which arise from competition of multiple polar phases^4,5^ induced by local chemical and structural inhomogeneity^6–9^. This nanoscale disorder, often attributed to cation disorder, gives rise to the hallmark of relaxor FE behavior: a strong dependence of the dielectric constant on frequency (dielectric dispersion) tied to evolution of polar states with temperature^10–13^, in contrast to weak frequency dispersion reported in standard FE solid solutions that is attributed to domain wall and phase boundary effects^14^. The degree of compositional ordering and the size of the resulting polar domains further modulates relaxor behavior^15^. For instance, the 1:1 type FE relaxors Pb(Sc_1/2_Nb_1/2_)O_3_ and Pb(Sc_1/2_Ta_1/2_)O_3_ can be either a classic or relaxor FE solely depending on the extent of *B*-site cation ordering, which can be kinetically controlled by annealing^15–18^. Numerous models of relaxor behavior have been proposed^8,19–21^ which are all based on the concept that the long-range ordering of polar domains must be limited by nanoscale disorder, while no ordered systems exhibiting relaxor behavior have been reported to date.

Here, we demonstrate that relaxor behavior can be realized without compositional randomness, owing to phase competition between the polar and antipolar states in a long-range cation-ordered perovskite. We stabilize highly cation-ordered films and demonstrate the coexistence of antiferroelectric (AFE) and FE phases in perovskite PbMg_0.5_W_0.5_O_3_. Using density functional theory (DFT), we identify a novel FE phase that is less than 1 meV/atom above the ground state AFE phase and is stabilized through compressive strain. The observed frequency-dependent dielectric relaxation correlates with the formation of mixed FE and AFE phases arising from compressive strain caused by the cooling substrate and the kinetic trapping of these phases at low temperatures. Our results show that relaxor behavior can be obtained in an elegantly simple material system distinguished by chemical composition homogeneity—paving the way for completely new relaxor FE materials that are not constrained by the limitations of the atomic disorder associated with conventional relaxor FEs.

## Results

### Structural characterization and *B*-site cation ordering in PbMg_0.5_W_0.5_O_3_

**Fig. 1:**
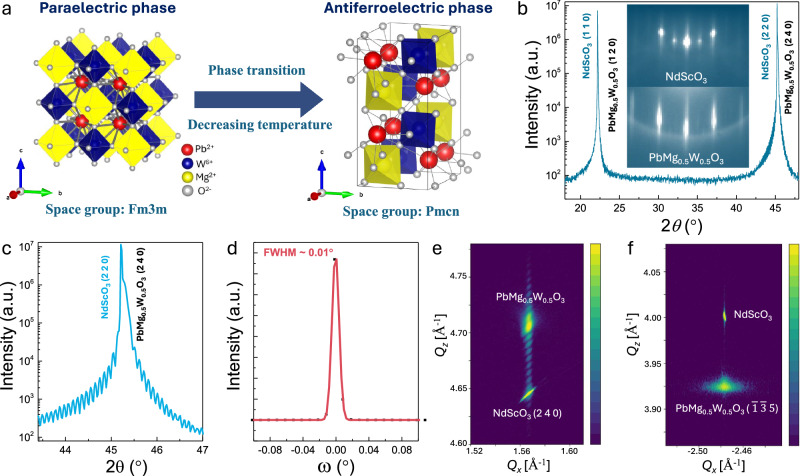
Thin film growth and X-ray diffraction (XRD) characterization of *B*-site ordered PbMg_0.5_W_0.5_O_3_ on NdScO_3_ substrate. **a** Atomic structure models of PbMg_0.5_W_0.5_O_3_ with PE phase (cubic) and AFE phase (orthorhombic). **b** Representative XRD *θ*−2*θ* scan of 100 nm-thick PbMg_0.5_W_0.5_O_3_/NdScO_3_ heterostructure. Inset: RHEED patterns of PbMg_0.5_W_0.5_O_3_ and NdScO_3_. **c** A higher resolution plot of the PbMg_0.5_W_0.5_O_3_ 024-diffraction condition with clear Laue oscillations. **d** The rocking curve of the PbMg_0.5_W_0.5_O_3_ (024) plane with FWHM of 0.01°. **e** RSM of NdScO_3_ 240-diffraction condition, exhibiting coherent epitaxial growth without strain relaxation. **f** RSM of PbMg_0.5_W_0.5_O_3_ $\bar{1}\,\bar{3}$ 5-diffraction, indicating *B*-site cation ordering.

Bulk PbMg_0.5_W_0.5_O_3_ has a double-perovskite (rocksalt) cubic structure and undergoes a first-order phase transition from cubic structure with the *F*m3m space group (paraelectric phase) to orthorhombic structure with the *P*mcn (*P*nma) space group (AFE phase) at *T*≈311 K, in close proximity to room temperature^22–28^ (Fig. 1a). Due to the large charge difference between Mg^2+^ and W^6+^, PbMg_0.5_W_0.5_O_3_ is well-known to display long-range rocksalt *B*-site cation ordering^27–30^ regardless of the synthesis protocol, in contrast with the Pb(Sc_1/2_Nb_1/2_)O_3_ and Pb(Sc_1/2_Ta_1/2_)O_3_ systems for which the smaller charge difference of 2 between Sc^3+^ and Nb/Ta^5+^ leads to the absence (presence) of long-range *B*-site cation ordering for samples obtained by quenching (annealing).

The high-quality epitaxial PbMg_0.5_W_0.5_O_3_ thin films were deposited by pulsed laser deposition (“Methods”). The 100-nm-thick PbMg_0.5_W_0.5_O_3_ film was grown on an NdScO_3_ (110) substrate with an excellent lattice matching and a representative x-ray diffraction *θ*−2*θ* scan (Fig. 1b) indicates the films are single-phase and epitaxial. The reflection high-energy electron diffraction (RHEED) patterns of the NdScO_3_ and PbMg_0.5_W_0.5_O_3_ film (inset of Fig. 1b), the surface morphology with very low roughness measured by atomic force microscopy (Fig. S1), and clear Laue oscillations (used to determine film thickness) of PbMg_0.5_W_0.5_O_3_ 024-diffraction conditions (Fig. 1c), indicate the two-dimensional (2D) growth mode, a smooth film surface and film-substrate interface. The full width at half maximum (FWHM) of rocking curves of the PbMg_0.5_W_0.5_O_3_ (024) plane is about 0.01° (Fig. 1d), indicating the high crystallinity and lateral coherence of PbMg_0.5_W_0.5_O_3_ films. The in-plane strain state of PbMg_0.5_W_0.5_O_3_/NdScO_3_ heterostructure, analyzed with reciprocal space mapping (RSM) of NdScO_3_ (240) (Fig. 1e), shows that the PbMg_0.5_W_0.5_O_3_ film is coherently strained to the substrates without strain relaxation and exhibits clear Laue oscillations. The appearance of PbMg_0.5_W_0.5_O_3_ $\bar{1}\bar{3}5$-reflection with *d*-spacing of 1.353 Å indicates the presence of the expected long-range *B*-site cation ordering (Fig. 1f).

**Fig. 2:**
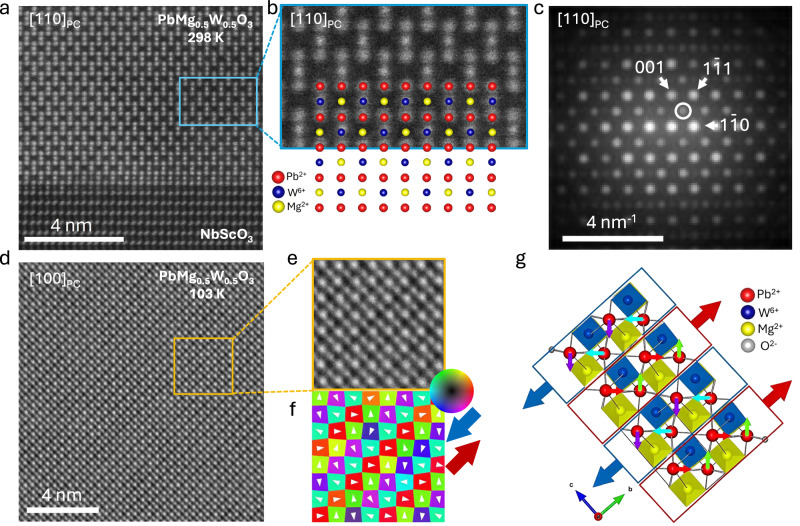
STEM characterization of PbMg_0.5_W_0.5_O_3_ thin films. **a** STEM-HAADF image of the PbMg_0.5_W_0.5_O_3_ film interface along the pseudocubic <110> projection taken at 298 K. **b** A magnified subregion is overlaid with the projected atomic model with Pb, Mg, and W cations. Mg (*Z* = 12) and O (*Z* = 8) are not visible, while Pb (*Z* = 82) and W (*Z* = 74) are clearly defined. **c** STEM-NBED pattern averaged from the film region along the pseudocubic <110> projection. The ½ $\left\{111\right\}$ superlattice reflection is circled and consistent with the rock-salt ordered structure. **d** Cryogenic STEM-HAADF image along the pseudocubic <100> projection taken at 103 K. **e** The subregion and its corresponding (**f**) ordered Pb vs. Mg/W cation displacements are consistent with AFE structure. The color wheel saturates at a displacement magnitude of 25 pm. **g** Atomic model of PMW, with AFE displacement pattern indicated and matching experiment.

To further reveal the atomic-level cation-ordered structure and antipolar Pb displacements, we performed scanning transmission electron microscopy (STEM) characterization at both ambient (298 K) and liquid nitrogen (approximately 103 K) temperatures. Viewed along the pseudocubic NdScO_3_ [1–10] (Fig. 2a) and [100] (Fig. S2) zone axis at room temperature, the cross-sectional high-angle annular dark-field (HAADF) STEM image of a 200-nm-thick PbMg_0.5_W_0.5_O_3_/NdScO_3_ film reveals alternating atom column intensity on the *B*-site sub-lattice. This atomic-number sensitive HAADF contrast is consistent with the expected ordering of Mg and W, as indicated by the overlaid atomic model (Fig. 2b). In addition, the nano-beam electron diffraction (NBED) pattern collected across a larger region of the film shows defined ½ $\left\{111\right\}$-type superlattice reflections (marked by white circle in Fig. 2c), consistent with a doubling of the repeat unit associated with rock-salt ordering of Mg and W. Subsequently, a cross-sectional sample viewed along the pseudocubic [100] zone axis was cooled to liquid nitrogen temperature and imaged in situ to fully recover the AFE state^31^. From the HAADF STEM image, the relative displacements between Pb and Mg/W atomic columns were mapped, revealing the characteristic antiparallel cation displacements associated with the AFE structure (Fig. 2d–f). The displacement pattern in experiments is consistent with that of the atomic model for AFE PbMg_0.5_W_0.5_O_3_ (Fig. 2g), in which four unique Pb displacement directions comprise the antipolar [110]-aligned dipole planes, as shown in Fig. 2f.

### Temperature evolution of switching behavior

**Fig. 3:**
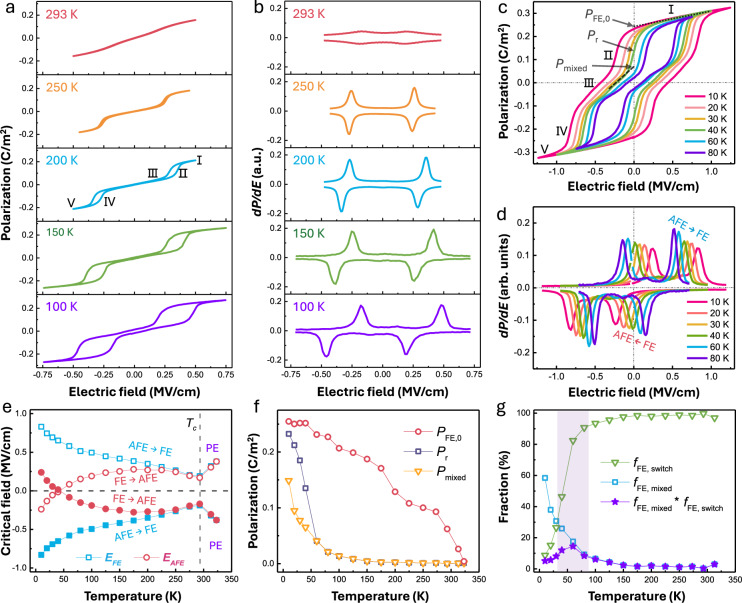
Temperature dependent characterization of polarization switching for PbMg_0.5_W_0.5_O_3_ thin films. **a**, **b** Polarization-electric field (*P*-*E*) loops and their derivative *dP*/*dE* vs electric field at the temperature of 293, 250, 200, 150, and 100 K, respectively. Extrapolation of the FE phase polarization to zero field is shown by dotted lines. **c** *P*-*E* loops and **d** *dP*/*dE* via electric field at the temperature from 10 to 80 K. **e** Temperature evolution of critical fields for the phase transition from AFE to FE and from FE to AFE extracted from temperature-dependent *P*-*E* loops. **f** Temperature evolution is shown for *P*_*r*_, *P*_FE,0_, and *P*_mixed_. **g** Temperature evolution of *f*_FE,mixed_, *f*_FE,switch_, and their product *f*_FE,mixed_* *f*_FE,switch_ representing the fraction of FE phase responsive to the applied *E* field.

To assess the electrical properties, Ba_0.6_Sr_0.4_RuO_3_ layers were deposited on NdScO_3_ as bottom electrodes (“Methods”) with the results of the corresponding structural characterizations of the PbMg_0.5_W_0.5_O_3_/Ba_0.6_Sr_0.4_RuO_3_/NdScO_3_ heterostructure displayed in Fig. S3. The out-of-plane polarization-electric-field (*P*-*E*) hysteresis loops and their derivative *dP/dE* plotted vs electric field of a 200-nm-thick PbMg_0.5_W_0.5_O_3_ film are presented in Fig. 3a, b for temperatures ranging from 293 to 100 K.

At *T* > 150 K, the hysteresis loops show classic AFE behavior where large polarization is observed at large positive *E* field and then shows five stages of decrease: slow, rapid, slow, rapid, and slow (Fig. 3a). The slow changes correspond to the changes in the intrinsic *P* of the two FE phases (Stages I and V) and the AFE phase (Stage III), while the rapid changes correspond to the switching of the material from FE to AFE and from AFE to FE (Stages II and IV). As the temperature decreases, the hysteresis loops become wider with greater difference in the *E*-fields of the AFE→FE (*E*_AFE→FE_) and FE→AFE (*E*_FE→AFE_) transitions (Supplementary Materials).

For *T* = 100 and 80 K, similar behavior is observed with five stages of decrease in *P* (Fig. 3c, d); however, a full switching from the FE phase to the AFE phase is not achieved and some amount of the FE phase remains even at zero field as indicated by non-zero remanent *P* (*P*_*r*_). Since at *E*_AFE→FE_ a kinetic barrier no longer prevents the transformation of the FE phase to the AFE phase, the retention of *P* at low *E* fields, including at zero field means that a mixed state comprised by both AFE and FE phases is thermodynamically preferred at these conditions with *P*_*r*_ equal to the polarization of the thermodynamically preferred mixed state at zero field (*P*_mixed_). The fraction of the FE phase in the mixed state at these temperatures can be estimated using the ratio between the zero-field *P* of the FE phase (*P*_FE,0_) and *P*_*r*_, where *P*_FE,0_ can be obtained by extrapolating the *E*-field dependence of *P* at high fields when the system is fully in the FE state (the dotted line in Fig. 3c). Due to the coexistence of the FE and AFE phases, the slow change of polarization in Stage III involves both the change in the intrinsic *P* of the AFE phase and the switching of the remaining FE phase from Stage II to the AFE phase. The temperature evolution of FE and AFE phases, and their apparent coexistence, is also revealed in the inelastic light scattering (Supplementary Materials), including the emergence of the cubic *T*_2g_(2) mode ≈ 65 cm^−1^ (Fig. S4) corresponding to antiphase vibration between *A*-site (Pb) and O octahedra, which splits into multiple lines at temperatures <100 K, similar to bulk^28^.

Comparison of the loops for 60 ≤ *T* ≤ 100 K (Fig. 3c) shows that as the temperature decreases, *P*_mixed_ is equal to *P*_*r*_ and increases strongly (Fig. 3f), indicating a rise in the fraction of the FE phase in the thermodynamically favored mixed phase. For *T* < 60 K, the transition to the thermodynamically preferred mixed state only occurs at negative *E* fields, indicating that the FE phase is kinetically trapped at *E* = 0. For these temperatures, *P*_mixed_ can be estimated by the extrapolation of the *P* dependence in Stage III to *E* = 0 (the dashed line in Fig. 3c). Thus, at these temperatures, the kinetic trapping of the FE phase becomes important, leading to wider FE loops and inability of the FE phase to switch to the AFE phase at zero field.

To quantitatively describe the changes in the switching characteristics of the PbMg_0.5_W_0.5_O_3_ films, we plot the characteristic parameters of the loops in Fig. 3e–g. A plot of *E*_AFE→FE_ and *E*_FE→AFE_ versus temperature (Fig. 3e) shows that for *T* > 250 K, *E*_AFE→FE_ = *E*_FE→AFE_, whereas for *T* < 250 K, *E*_AFE→FE_ exhibits the expected behavior of rising with decreasing temperature while *E*_FE→AFE_ first increases and then starts to decrease at ≈150 K, due to the increasingly strong stabilization of the FE phase. The plot of *P*_*r*_, *P*_mixed_ and *P*_FE,0_ (Fig. 3f) shows that *P*_FE,0_ exhibits a gradual decline from 0.255 C m^−2^ at 10 K to ~0 at 300 K, while *P*_*r*_ decreases rapidly from 0.233 C m^−2^ at 10 K to 0.04 C m^−2^ at 60 K and then decreases slowly to 0 at 150 K. Based on the values of *P*_FE,0_, *P*_*r*_, and *P*_mixed_ we can estimate the fraction of PbMg_0.5_W_0.5_O_3_ films that is thermodynamically preferred to be in the FE phase at *E* = 0 (*f*_FE,mixed_ = *P*_mixed_/*P*_FE,0_), the fraction of the FE phase that is switchable to the AFE phase at *E* = 0 (*f*_FE,switch_ = (*P*_FE,0_–*P*_*r*_)/*P*_FE,0_). The total amount of the FE phase that can be switchable in a film at thermodynamic equilibrium (non-poled) at zero-field can be estimated as the product of *f*_FE,mixed_ and *f*_FE,switch_ (Fig. 3g). It can be seen *f*_FE,mixed_*f_FE,switch_ starts rising at 125 K, peaks at 60 K and then decreases to zero at 10 K. This suggests that the response to the applied *E* field will depend most strongly on the *E* field frequency in the region at ~60 K. At higher temperatures, the switching is very fast due to low kinetic barriers at *E* = 0, whereas at lower temperature the system is kinetically trapped by a very high barrier so that switching does not occur on the timescale of the hysteresis loop measurements.

### Relaxor behavior in PbMg_0.5_W_0.5_O_3_

**Fig. 4:**
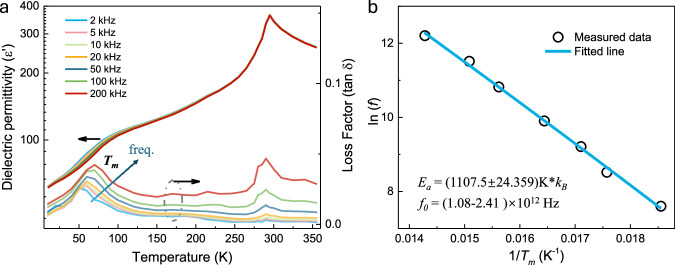
Dielectric relaxation of PbMg_0.5_W_0.5_O_3_ thin films. **a** Dielectric permittivity and loss factor as a function of temperature and frequency (2–200 kHz). **b** The frequency dependence of *T*_m_ for PbMg_0.5_W_0.5_O_3_ films. *T*_m_ is the temperature for the maximum loss factor for a specific frequency.

To show the relaxor characteristics, we examine the temperature-dependent dielectric permittivity (*ɛ*´) and loss (tan*δ*) at various frequencies (Fig. 4a). As the temperature decreases, a dielectric enhancement without frequency dispersion is observed near room temperature, corresponding to a sharp first-order phase transition between PE and AFE phases (Fig. 1a) at the Curie temperature (*T*_c_). As the temperature is reduced to around 130 K, a distinct dielectric relaxation behavior is identified, where the drop in permittivity and a concomitant peak in the dielectric loss demonstrate a large variation as a function of driving frequency, similar to that observed in relaxor FEs^11,12^. The relationship between the temperature of maximum dielectric loss (*T*_m_) of the low-temperature relaxor phase and the frequency (*f*) can be described by Arrhenius law^11^:

1 $$f={f}_{0}\exp \left[-{E}_{{{\rm{a}}}}/{k}_{{{\rm{B}}}}{T}_{{{\rm{m}}}}\right]$$

where *f* is the frequency of measurement, *f*_0_ is the attempt jump frequency for dipoles, *E*_a_ is the activation energy, and *k*_B_ is the Boltzmann constant. The linear relationship between ln (*f*) and 1/*T*_m_ and the fitted parameters using Eq. (1) are shown in Fig. 4b. *E*_a_ ≈ 0.095 eV is similar to the activation energy in Pb-relaxors^11,32,33^. However, in contrast to Pb(Mg_1/3_Nb_2/3_)O_3_-PbTiO_3_, the relaxation cannot be attributed to the weak coupling at Mg-rich local environments^8^ due to the full *B*-cation order in the PbMg_0.5_W_0.5_O_3_ films. The similarity between the temperature of the onset of the relaxor behavior and the temperature at which *E*_AFE_ starts to deviate from the expected monotonic temperature evolution and *P*_*r*_ begins to increase suggest that both of these effects are caused by the same structural phenomenon that occurs at *T* ≈ 100–130 K. Furthermore, as observed from Fig. 4a, after the onset at 130 K, the difference between ε(*T*) at different frequencies first increases with lower *T* until *T* ≈ 60 K and then decreases. The temperature range between 60 and 130 K corresponds to the temperature range of rapid rise in *P*_mixed_ (FE phase stabilization) and weak kinetic trapping of the FE phase observed in the hysteresis loop, whereas the strong kinetic trapping of the FE phase corresponds to weaker dispersion observed for *T* < 60 K. This suggests that the relaxor behavior is due to the partial stabilization of the FE phase and coexistence of the FE and AFE phases; the coexistence of the phases breaks the long-range polar ordering and leads to the formation of nanopolar domains that respond differently to different frequencies of applied field. The maximum dispersion also corresponds to the maximum of *f*_FE,mixed_*f_FE,switch_ (purple shadow in Fig. 3g), suggesting that at these temperatures the FE and AFE phases achieve both relatively high mixing due to a high *f*_FE,mixed_ which creates a high density of FE/AFE interfaces/domain walls, similar to the high density of domain walls found in relaxor FEs while retaining the ability to respond to the applied *E*-field (high *f*_FE,switch_).

Defects such as oxygen vacancies often have complex effects on conventional FEs and in some cases their presence at high concentration gives rise to relaxor behavior as well as high leakage and asymmetric hysteresis loops^34,35^. In this work, to reduce the effects of defects, high-quality epitaxial films were obtained by optimizing growth conditions followed by post-annealing in a high-oxygen atmosphere. These films exhibit low leakage and symmetric hysteresis loops, indicating a low concentration of defects such as oxygen vacancies in the films. Such low concentrations of defects do not give rise to relaxor behavior in films of AFE materials such as PbZrO_3_. Therefore, it is unlikely that the observed relaxor behavior is due to the presence of oxygen vacancies and other defects, and rather arises from the competition between the AFE and FE phase as discussed above.

Relaxor dispersion in the vicinity of AFE-FE phase transition has been previously observed in Na₀.₅Bi₀.₅TiO₃ (NBT) and NaNbO₃; however, both of these systems are distinct from the PMW films examined in this study. NBT films show strong cation disorder on the A-site, so that relaxor behavior observed in this system is considered to arise from cation disorder, similar to the classic PMN material. While NaNbO₃ is nominally ion-disorder-free, it is a highly complex material that is known to be extremely sensitive to a wide variety of factors during synthesis, including small deviations from stoichiometry and homogeneity; thus, due to its extreme sensitivity, even low concentrations of defects may affect NaNbO₃ strongly enough to give rise to relaxor behavior. Furthermore, the FE and AFE phase in NaNbO₃ are due to the off-center displacements of O anions rather than the cation as is the case in PMW and other standard FEs such as PMN and PZT. Therefore, the relaxor behavior in NaNbO₃ can be considered to be a result of the unusual structural properties of NaNbO₃. Thus, due to these distinctions between our systems and the previously reported NBT and NaNbO₃, our work on high-quality, single-crystal PMW films demonstrates a new paradigm for designing relaxor behavior in cation-driven perovskite FEs through engineered phase competition.

**Fig. 5:**
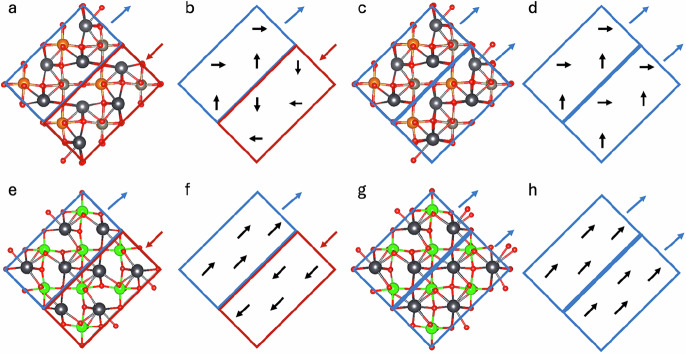
Comparison of antiferroelectric and ferroelectric atomic configurations in PbMg_0.5_W_0.5_O_3_ and PbZrO_3_. Most stable AFE (**a**, **b**) and FE (**c**, **d**) atomic configurations of bulk PbMg_0.5_W_0.5_O_3_, showing the atomic structures (**a**, **c**) and corresponding Pb displacement patterns (**b**, **d**). Most stable AFE (**e**, **f**) and FE (**g**, **h**) configurations of bulk PbZrO_3_, showing the atomic structures (**e**, **g**) and corresponding Pb displacement patterns (**f**, **h**). Arrows indicate the directions of Pb displacements.

To characterize the atomic configuration of this newly observed FE state, we used DFT calculations to find the most stable FE phase for bulk PbMg_0.5_W_0.5_O_3_. We found that instead of homogeneous displacement of Pb atoms along a single axis, this phase has a polarization along (110) generated by alternating Pb displacements along (100) and (010), similar to the AFE ground state. These results are quite different from the FE phase of the prototypical AFE PbZrO_3_, which shows a standard uniform displacement for all Pb and Zr ions (Fig. 5). Interestingly, the FE phase was found to be only 0.5 meV per atom higher in energy than the AFE phase for bulk PbMg_0.5_W_0.5_O_3_, while for PbZrO_3_ the energy difference is over 2 meV per atom. The small energy difference between the AFE and FE phases is consistent with the observation of the stabilization of the FE phase at low temperatures revealed by the hysteresis loops.

**Fig. 6:**
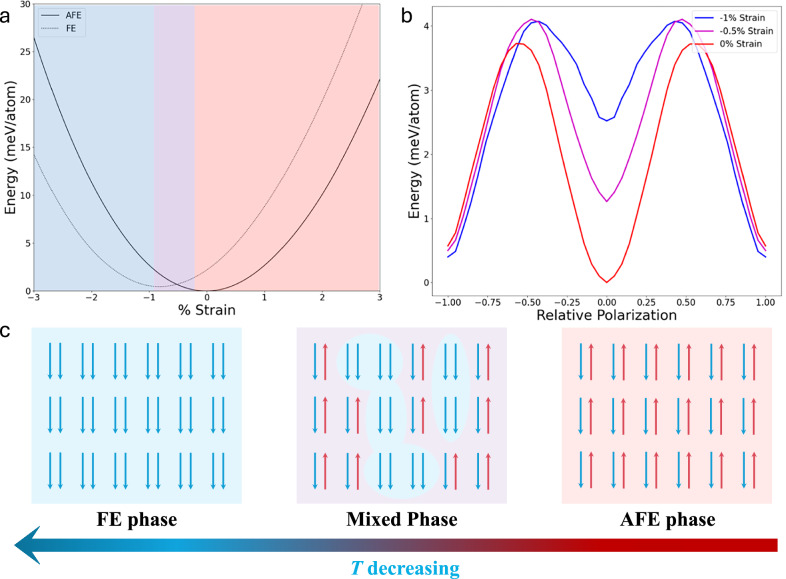
The influence of strain changes on the relative stabilities of the FE and AFE phases of PbMg_0.5_W_0.5_O_3_. **a** Energy for the AFE and FE phase as a function of strain from equilibrium PbMg_0.5_W_0.5_O_3_ cell. For any compressive modification, the FE phase is preferred (blue) while the opposite is true for AFE (red). There also exists a regime with a small compressive strain where the phase energy is almost indistinguishable (purple) and the phase energies remain close throughout. **b** Corresponding triple well potential for each of the stability regimes. When either phase is preferred, the other remains close in energy and the barrier for transitioning between them remains similar. **c** A schematic for the phase evolution and competition accounting for the relaxor behavior at the cryogenic temperature.

To shed light on the appearance of the FE phase at low temperatures (<150 K), we examine the influence of strain changes on the relative stabilities of the FE and AFE phases (Fig. 6). Strain changes will appear due to the contraction of the lattice for both substrate and PbMg_0.5_W_0.5_O_3_ upon cooling. Our calculations show that the two phases change thermodynamic ordering with strain, with the equilibrium volume of the FE phase being 1% smaller than that of the AFE phase for relaxations at 0 K (Fig. 6a). Calculations of both the phase energies and the homogeneous switching barriers (triple wells) at different strain states show that compressive strain promotes the formation of the FE phase, with the FE phase becoming the ground state with a moderate (≈ −0.3%) change in the strain (Fig. 6b) similar to the strain imposed by the substrate on PbMg_0.5_W_0.5_O_3_ at low temperatures^36,37^ without strain relaxations (Fig. S5 and Supplementary Materials). This suggests that the FE phase will be stabilized by the shrinkage of the lattice parameters of the substrates during cooling, consistent with the appearance of the FE phase at *T* < 150 K observed from the measured hysteresis loops.

We then performed calculations for the intrinsic FE→AFE switching pathway; here as well, we found that PbMg_0.5_W_0.5_O_3_ exhibits unusual behavior where dipoles must only rotate 90° (Fig. S6) to change the polarization by 180°, leading to much lower switching energy, which accounts for the lower *E*_AFE_ and *E*_FE_ of PbMg_0.5_W_0.5_O_3_ compared to that of the typical AFE PbZrO_3_.

Figure 6c depicts the schematic of structural evolution associated with relaxor behavior as a function of temperature. At higher temperature, the AFE phase is energetically favorable. With decreasing temperature, the energy difference between the FE and AFE phases decreases and the long-range antipolar ordering is disrupted by the formation of small regions of FE phase (likely at the AFE domain walls) intermixed with the regions of the AFE phase, resulting in a diffuse phase transition behavior at the intermediate temperature. As temperature is further reduced, the energy difference between FE and AFE phases decreases further, and the fraction of the FE polar phase with non-zero macroscopic polarization grows further, but the FE phase is kinetically trapped and cannot switch in response to the applied *E*-field.

Further insights into the connection between the AFE-FE phase competition and observed relaxor behavior would benefit from theoretical modeling of relaxor dynamics in PMW. However, simple models for understanding relaxor dynamics are currently lacking, while MD simulations of coexisting AFE and FE phases in PMW would be highly non-trivial and require parameterization and validation of atomistic potentials as well as simulations at large length- and timescales. Such simulations are outside the scope of this work and will be conducted in future research.

While compositional inhomogeneity and the presence of nanoscale regions with short-range structural correlations have long been believed to be two primary prerequisites for a relaxor, our work shows that the competition between the FE and AFE phases can lead to relaxor behavior even in a fully ordered oxide. The thermodynamically determined local polar development associated with (anti)ferroelectric phase competition is promising for realizing and harnessing relaxor behavior without compositional inhomogeneity, akin to the intrinsic phase competition that enables unexpected responses in lattice and charge–orbital ordered antiferromagnetics^38^ and its temperature evolution of magnetic hysteresis across magnetic phases^39^.

## Methods

### Sample preparation

Heterostructures of 100 nm-PbMg_0.5_W_0.5_O_3_/NdScO_3_ and 30 nm-SrRuO_3_/200 nm- PbMg_0.5_W_0.5_O_3_/20 nm-Ba_0.6_Sr_0.4_RuO_3_/NdScO_3_ were synthesized via pulsed-laser deposition, with a target-to-substrate distance of 5.5 cm, using a KrF excimer laser (248 nm, Coherent). The Ba_0.6_Sr_0.4_RuO_3_ bottom electrodes were grown at a heater temperature of 780 °C in a dynamic oxygen pressure of 20 mTorr with a laser fluence of 2 J cm^−2^ and a laser repetition rate of 3 Hz from a ceramic target of the same composition. The PbMg_0.5_W_0.5_O_3_ layers were grown at a heater temperature of 580 °C in a dynamic oxygen pressure of 200 mTorr with a laser fluence of 2 J cm^−2^ and a laser repetition rate of 2 Hz from a ceramic target of the same composition with 10% lead excess fabricated by solid state reaction to compensate for lead loss during growth. The SrRuO_3_ top electrode layers were grown in situ immediately following PbMg_0.5_W_0.5_O_3_ deposition at a heater temperature of 530 °C in a dynamic oxygen pressure of 100 mTorr with a laser fluence of 2 J cm^−2^ and a laser repetition rate of 6 Hz. Following deposition, the heterostructures were cooled at a rate of 5 °C min^−1^ in a static oxygen pressure of 300 Torr. Whole growth process is monitored in situ by reflection high-energy electron diffraction (RHEED). The capacitor structures were fabricated via standard photolithography process and wet etching lithography. The top SrRuO_3_ layer was selectively wet etched by an NaIO_4_ aqueous solution (0.1 mol L^−1^) to realize circular capacitor structures with a diameter of 25 μm.

### X-ray diffraction (XRD) measurements

The room-temperature XRD *θ*−2*θ* scans along a normal direction, rocking curves (ω scans) of PbMg_0.5_W_0.5_O_3_ (024), and 2D RSM were studied using Malvern Panalytical Empyrean XRD system (PANalytical), with Cu Kα (wavelength ≈ 1.5406 Å) X-ray.

### Scanning probe microscopy

Surface topography of the PMW films was evaluated by scanning probe microscopy (Asylum Research Cypher, Oxford Instruments) using intermittent contact mode.

### Electrical measurements

The *P*-*E* hysteresis loops were measured at a frequency of 10 kHz using a Precision Multiferroic Tester (Radiant Technologies) in a probe station. Dielectric response of the heterostructures was measured as a function of frequency (2–200 kHz) using an impedance analyzer (Keysight E4990A). The temperature of electrical measurement is from 10 to 350 K.

### Electron microscopy

Cross-section samples for STEM characterization were prepared by Ga+ focused ion beam (Thermo Fisher Scientific Helios 600) lift out. Thinning to electron transparency was achieved by milling with decreasing Ga+ ion beam energy from 30 to 5 kV. The final thinning to remove Ga+ damage was performed by broad beam Ar+ ion milling (Fischione 1051 TEM Mill) using beam energies of 0.3 and 0.1 kV. Electron microscopy datasets were acquired using a probe aberration-corrected STEM (Thermo Fisher Scientific Themis-Z S/TEM) at an accelerating voltage of 300 kV with probe semi-convergence angle of 21.8 mrad and beam current of ≈10 pA. For in situ cryo-STEM imaging, cross-sectional lamellae were prepared by focused ion beam lift out and mounted on micro-electromechanical chips (DENSsolutions), after which cooling to liquid nitrogen temperature was performed using a dedicated cryogenic cooling holder (DENSsolutions Arctic Lightning).

### Raman scattering spectroscopy

Raman scattering spectroscopy was collected at selected temperatures between 1.6 and 350 K (OptiCool, Quantum Design) in the backscattering geometries $Z({YY})\bar{Z}$ (parallel) and $Z({XY})\bar{Z}$ (crossed) under 325 nm HeCd laser excitation (Kimmon-Koha), $\approx \,$ mW, focused to a ≈20 μm spot diameter, using a triple subtractive spectrometer (T64000, Horiba Jobin-Yvon) and an air-cooled electron-multiplying charged-coupled detector camera (Andor Newton). The sample was first cooled to 10 K and then spectra were collected from 10 to 100 K in 10 K steps, and from 100 to 350 K in 25 K steps.

### Theoretical calculations

A full switching from the FE phase to the AFE phase is not achieved and some amount of the FE phase remains even at zero field as indicated by non-zero remanent *P* (*P*_*r*_). *P*_FE,0_ can be obtained by extrapolating the *E*-field dependence of *P* at high fields when the system is fully in the FE state (the dotted line in Fig. 3c). *P*_mixed_ can be estimated by the extrapolation of the *P* dependence in Stage III to *E* = 0 (the dashed line in Fig. 3c). DFT and NEB calculations were performed using the Quantum Espresso^40^ code with optimized norm-conserving pseudopotentials generated with the OPIUM^41,42^ code. All calculations were performed at 0 K. The generalized gradient approximation of Perdew, Burke, and Ernzerhof was used to calculate the exchange correlation energy^43^. Convergence criteria of 1.4e-5 eV per cell and 2.6e-4 eV Å^−1^ for energy and forces, respectively, were used for all calculations.

## Supplementary information

Supplementary Information

Transparent Peer Review file

## Data Availability

The datasets generated during and/or analysed during the current study are available in the figshare repository, https://figshare.com/s/ef43d93cb5d3725b2452.

## References

[1] Li, F. et al. Giant piezoelectricity of Sm-doped Pb(Mg_1/3_Nb_2/3_)O_3_-PbTiO_3_ single crystals. *Science* **364**, 264–268 (2019). 31000659 10.1126/science.aaw2781

[2] Pan, H. et al. Ultrahigh–energy density lead-free dielectric films via polymorphic nanodomain design. *Science* **365**, 578–582 (2019). 10.1126/science.aaw8109 31395780

[3] Li, F. et al. Ultrahigh piezoelectricity in ferroelectric ceramics by design. *Nat. Mater.* **17**, 349–354 (2018). 29555999 10.1038/s41563-018-0034-4

[4] Damjanovic, D. Comments on origins of enhanced piezoelectric properties in ferroelectrics. *IEEE Trans. Ultrason. Ferroelectr. Freq. Control* **56**, 1574–1585 (2009). 19686973 10.1109/TUFFC.2009.1222

[5] Fu, H. & Cohen, R. E. Polarization rotation mechanism for ultrahigh electromechanical response in single-crystal piezoelectrics. *Nature* **403**, 281–283 (2000). 10659840 10.1038/35002022

[6] Li, F., Zhang, S., Damjanovic, D., Chen, L. Q. & Shrout, T. R. Local structural heterogeneity and electromechanical responses of ferroelectrics: learning from relaxor ferroelectrics. *Adv. Funct. Mater.* **28**, 1801504 (2018).

[7] Krogstad, M. J. et al. The relation of local order to material properties in relaxor ferroelectrics. *Nat. Mater.* **17**, 718–724 (2018). 29941922 10.1038/s41563-018-0112-7

[8] Takenaka, H., Grinberg, I., Liu, S. & Rappe, A. M. Slush-like polar structures in single-crystal relaxors. *Nature* **546**, 391–395 (2017). 28617453 10.1038/nature22068

[9] Kumar, A. et al. Atomic-resolution electron microscopy of nanoscale local structure in lead-based relaxor ferroelectrics. *Nat. Mater.* **20**, 62–67 (2021). 32895506 10.1038/s41563-020-0794-5

[10] Gao, X. et al. The mechanism for the enhanced piezoelectricity in multi-elements doped (K,Na)NbO_3_ ceramics. *Nat. Commun.* **12**, 881 (2021). 33564001 10.1038/s41467-021-21202-7 PMC7873261

[11] Li, F., Zhang, S., Xu, Z. & Chen, L.-Q. The contributions of polar nanoregions to the dielectric and piezoelectric responses in domain-engineered relaxor-PbTiO_3_ Crystals. *Adv. Funct. Mater.* **27**, 1700310 (2017).

[12] Li, F. et al. The origin of ultrahigh piezoelectricity in relaxor-ferroelectric solid solution crystals. *Nat. Commun.* **7**, 13807 (2016). 27991504 10.1038/ncomms13807 PMC5187463

[13] Cowley, R. A., Gvasaliya, S. N., Lushnikov, S. G., Roessli, B. & Rotaru, G. M. Relaxing with relaxors: a review of relaxor ferroelectrics. *Adv. Phys.* **60**, 229–327 (2011).

[14] Kersten, O. & Schmidt, G. Dielectric dispersion in PZT ceramics. *Ferroelectrics* **67**, 191–197 (1986).

[15] Setter, N. & Cross, L. E. The role of B-site cation disorder in diffuse phase transition behavior of perovskite ferroelectrics. *J. Appl. Phys.* **51**, 4356–4360 (1980).

[16] Perrin, C. et al. Influence of B-site chemical ordering on the dielectric response of the Pb(Sc_1/2_Nb_1/2_)O_3_ relaxor. *J. Phys. Condens. Matter* **13**, 10231 (2001).

[17] Bokov, A. A. & Rayevsky, I. P. Recent advances in compositionally orderable ferroelectrics. *Ferroelectrics* **144**, 147–156 (1993).

[18] Bidault, O., Perrin, C., Caranoni, C. & Menguy, N. Chemical order influence on the phase transition in the relaxor Pb(Sc_1/2_Nb_1/2_)O_3_. *J. Appl. Phys.* **90**, 4115–4121 (2001).

[19] Eremenko, M. et al. Local atomic order and hierarchical polar nanoregions in a classical relaxor ferroelectric. *Nat. Commun.* **10**, 2728 (2019). 31227698 10.1038/s41467-019-10665-4 PMC6588601

[20] Fu, D. et al. Relaxor Pb(Mg_1/3_Nb_2/3_)O_3_: a ferroelectric with multiple inhomogeneities. *Phys. Rev. Lett.* **103**, 207601 (2009). 20366012 10.1103/PhysRevLett.103.207601

[21] Glazounov, A. E., Tagantsev, A. K. & Bell, A. J. Evidence for domain-type dynamics in the ergodic phase of the PbMg_1/3_Nb_2/3_O_3_ relaxor ferroelectric. *Phys. Rev. B* **53**, 11281–11284 (1996). 10.1103/physrevb.53.11281 9982731

[22] Baldinozzi, G., Sciau, P., Pinot, M. & Grebille, D. Crystal structure of the antiferroelectric perovskite Pb2MgWO6. *Acta Crystallogr. Sect. B Struct. Sci.* **51**, 668–673 (1995).

[23] Yang, J. H., Kim, H. J., Choo, W. K. & Lee, C. T. Antiferroelectric superstructures of Pb_2_MgWO_6_. *Ferroelectrics* **152**, 243–248 (1994).

[24] Baba-Kishi, K. Z., Cressey, G. & Cernik, R. J. X-ray and electron diffraction studies of the structures of pseudo-perovskite compounds Pb_2_(Sc,Ta)O_6_ and Pb_2_(Mg,W)O_6_. *J. Appl. Crystallogr.* **25**, 477–487 (1992).

[25] Yasuda, N., Fujimoto, S. & Yoshimura, T. Pressure and temperature dependence of dielectric properties of Pb(Mg_1/2_W_1/2_)O_3_. *J. Phys. C: Solid State Phys.* **19**, 1055 (1986).

[26] Smolenskii, G. A., Agranovskaya, A. I. & Isupov, V. A. New ferroelectrics of complex composition: Pb_2_MgWO_6_, Pb_3_Fe_2_WO_9_, and Pb_2_FeTaO_6_. *Sov. Phys. Solid State* **1**, 907–908 (1959).

[27] Flerov, I. N., Gorev, M. V. & Ph, S. Heat capacity and p-T phase diagrams of the ordered perovskites Pb_2_MgWO_6_ and Pb_2_CoWO_6_. *J. Phys. Condens. Matter* **12**, 559–567 (2000).

[28] Kania, A., Jahfel, E., Kugel, G. E., Roleder, K. & Hafid, A. M. A Raman investigation of the ordered complex perovskite PbMg_0.5_W_0.5_O_3_. *J. Phys.: Condens. Matter* **8**, 4441–4453 (1996.

[29] Baldinozzi, G., Sciaut, P. & Bulou, A. Raman study of the structural phase transition in the ordered perovskite Pb_2_MgWO_6_. *J. Condens. Matter Phys.* **7**, 8109–8117 (1995).

[30] Choo, W. K. et al. Crystal structure and B-site ordering in antiferroelectric Pb(Mg_1/2_W_1/2_)O_3_, Pb(Co_1/2_W_1/2_)O_3_ and Pb(Yb_1/2_Nb_1/2_)O_3_. *JPN J. Appl. Phys.* **32**, 4249–4253 (1993).

[31] Zhu, M. et al. Insights into chemical and structural order at planar defects in Pb_2_MgWO_6_ using multislice electron ptychography. *ACS Nano* **19**, 5568–5576 (2025). 39871489 10.1021/acsnano.4c14833

[32] Pramanick, A. & Nayak, S. Perspective on emerging views on microscopic origin of relaxor behavior. *J. Mater. Res.* **36**, 1015–1036 (2021).

[33] Stock, C. et al. Interplay between static and dynamic polar correlations in relaxor Pb(Mg_1/3_Nb_2/3_)O_3_. *Phys. Rev. B* **81**, 144127 (2010).

[34] Chatterjee, S. et al. Role of oxygen vacancies on the low-temperature dielectric relaxor behavior in epitaxial Ba_0.85_Ca_0.15_Ti_0.9_Zr_0.1_O_3_ thin films. *Phys. Rev. Mater.* **5**, 064415 (2021).

[35] Deng, G., Li, G., Ding, A. & Yin, Q. Evidence for oxygen vacancy inducing spontaneous normal-relaxor transition in complex perovskite ferroelectrics. *Appl. Phys. Lett.* **87**, 192905 (2005).

[36] Uecker, R. et al. Properties of rare-earth scandate single crystals (Re=Nd−Dy). *J. Cryst. Growth* **310**, 2649–2658 (2008).

[37] Baldinozzi, G., Sciau, P. & Buffat, P.-A. Investigation of the orthorhombic structures of Pb_2_MgWO_6_ and Pb_2_CoWO_6_. *Solid State Commun.* **86**, 541–544 (1993).

[38] Rairigh, R. P. et al. Colossal magnetocapacitance and scale-invariant dielectric response in phase-separated manganites. *Nat. Phys.* **3**, 551–555 (2007).

[39] Matsuura, K. et al. Thermodynamic determination of the equilibrium first-order phase-transition line hidden by hysteresis in a phase diagram. *Sci. Rep.* **13**, 6876 (2023). 37106004 10.1038/s41598-023-33816-6 PMC10140377

[40] Giannozzi, P. et al. QUANTUM ESPRESSO: a modular and open-source software project for quantum simulations of materials. *J. Phys. Condens. Matter* **21**, 395502 (2009). 21832390 10.1088/0953-8984/21/39/395502

[41] Rappe, A. M., Rabe, K. M., Kaxiras, E. & Joannopoulos, J. D. Optimized pseudopotentials. *Phys. Rev. B Condens. Matter* **41**, 1227–1230 (1990). 9993827 10.1103/physrevb.41.1227

[42] Ramer, N. J. & Rappe, A. M. Designed nonlocal pseudopotentials for enhanced transferability. *Phys. Rev. B* **59**, 12471–12478 (1999).

[43] Perdew, J. P., Burke, K. & Ernzerhof, M. Generalized gradient approximation made simple. *Phys. Rev. Lett.* **77**, 3865–3868 (1996). 10062328 10.1103/PhysRevLett.77.3865

