# Supplementary material for: Relaxor behavior in rocksalt cation-ordered material induced by (anti)ferroelectric phase competition
Source: Nat Commun. 2026 Jun 23;17:7566. doi: 10.1038/s41467-026-74237-z (PMC13416127; doi:10.1038/s41467-026-74237-z)
Supplement: Supplementary file 1 — Supplementary Information [file 41467_2026_74237_MOESM1_ESM.pdf]

Supplementary Information

Relaxor behavior in rocksalt cation-ordered material induced by (anti)ferroelectric phase competition

Yu Yun[1,§], Liyan Wu[1,§,*], Drew Behrendt[2,§], Hao Pan[3,§], Menglin Zhu[4], Michael Xu[4], Anthony J. Ruffino[1], John Carroll[5], Irina Baraban[6], Zishen Tian[3], Xianfei Xu[2], Dongfang Chen[1], Brendan M. Hanrahan[7], Lane W. Martin[3,8,9], James M. LeBeau[4], Andrew M. Rappe[2], Ilya Grinberg[10] and Jonathan E. Spanier[1,5,6,11,*]

[1]Department of Mechanical Engineering and Mechanics, Drexel University, Philadelphia, PA, 19104 USA
[2]Department of Chemistry, University of Pennsylvania, Philadelphia, PA, 19104 USA
[3] Department of Materials Science and Engineering, University of California, Berkeley, CA, 94720 USA
[4]Department of Materials Science & Engineering, MIT, Cambridge, MA, 02139 USA
[5]Department of Electrical and Computer Engineering, Drexel University, Philadelphia, PA, 19104 USA
[6]Department of Physics, Drexel University, Philadelphia, PA, 19104 USA
[7]DEVCOM, Army Research Laboratory, Adelphi, MD, 20783 USA
[8]Departments of Materials Science and Nanoengineering, Chemistry, and Physics and Astronomy, Rice University, Houston, TX, 77005 USA
[9]Rice Advanced Materials Institute, Rice University, Houston, TX, 77005 USA
[10]Department of Chemistry, Bar-Ilan University, Ramat Gan, 5290002 Israel
[11]Department of Materials Science & Engineering, Drexel University, Philadelphia, PA, 19104 USA

[§]These authors contributed equally.

*Email: lw627@drexel.edu; spanier@drexel.edu

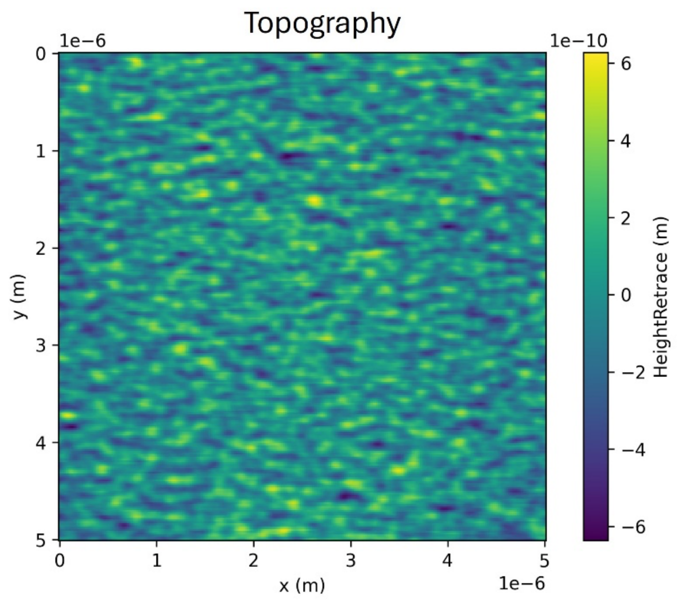

Fig. S1. Scanning probe microscopy height image. Scanning probe microscopy height image for 100-nm-thick $PbMg_{0.5}W_{0.5}O_3$ films with an area of 5 µm × 5 µm.

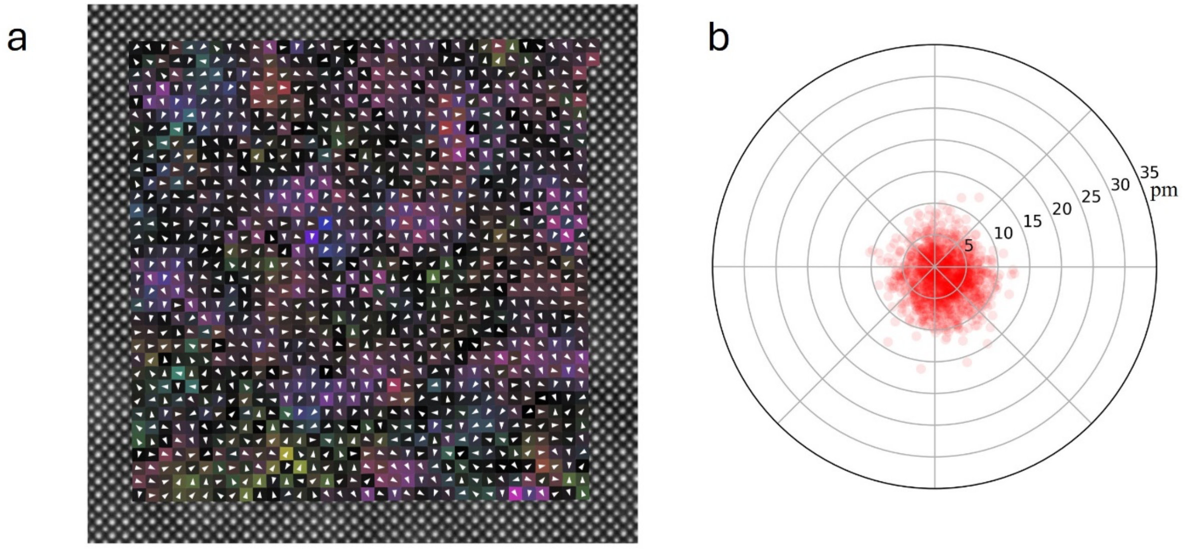

Fig. S2 Atomic resolution structure and cation displacements at room temperature. a. STEM image of $PbMg_{0.5}W_{0.5}O_3$ film at room temperature along the pseudocubic [100]-type projection with disordered cation displacements. **b**. Polar plot of the cation displacements from the image, in picometers.

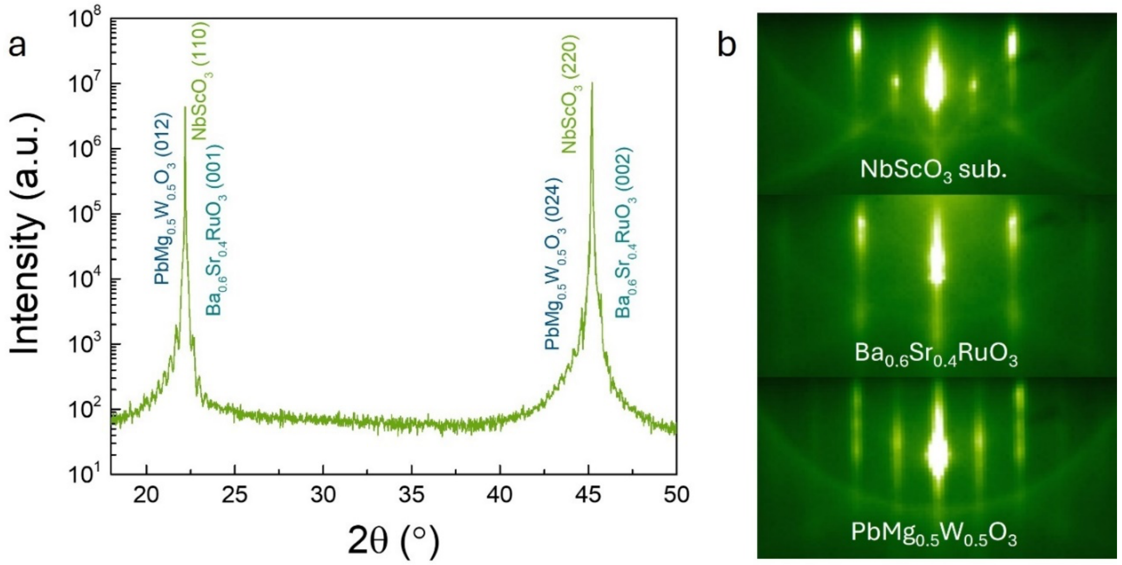

Fig. S3. Additional X-ray diffraction and reflection high-energy electron diffraction data. a. XRD θ-2θ scan and **b.** RHEED patterns of the $PbMg_{0.5}W_{0.5}O_3/Ba_{0.6}Sr_{0.4}RuO_3/NbScO_3$ heterostructure.

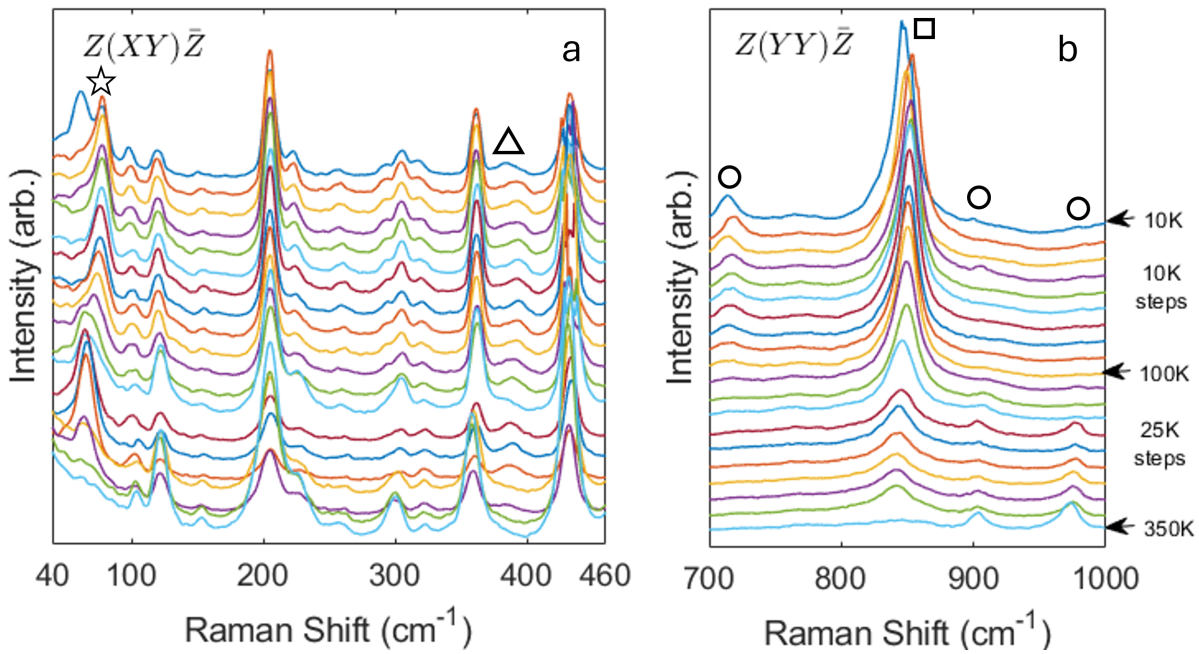

Fig. S4 Temperature evolution of the Raman scattering spectra for **a.** crossed and **b.** parallel polarization configurations, collected in the backscattering geometry. The $T_{2g}(2)$, $T_{2g}(1)$, and A_{1g} modes are denoted by star, triangle, and square, respectively. Modes denoted by a circle are discussed further, all other modes present are $NdScO_3$ substrate modes or minor unidentified modes of $PbMg_{0.5}W_{0.5}O_3$.

Temperature-dependent polarized Raman scattering spectroscopy and analysis of phase evolution

$PbMg_{0.5}W_{0.5}O_3$ bulk has a T_C of ≈313 K, below which it has an orthorhombic structure (*Pmcn*), and above a cubic structure (*Fm3m*). Bulk $PbMg_{0.5}W_{0.5}O_3$ has been characterized by Raman scattering spectroscopy both as a single crystal [SR1] and as a sintered powder [SR2] through a wide range of temperatures above and below T_C. The mode identification follows from the zone center irreducible representations (irreps), and the cubic phase irreps determined in [SR1] are as follows:

$$\Gamma(k_0) = A_{1g} \oplus E_g \oplus T_{1g} \oplus 2T_{2g} \oplus 5T_{1u} \oplus T_{2u}$$

The Raman-active modes are the A_{1g} mode and the two T_{2g} modes. The E_g mode, which would normally be Raman-active, has not been observed in the tungstates [SR1]. The A_{1g} and the two T_{2g} modes have been observed in bulk $PbMg_{0.5}W_{0.5}O_3$ from 10 K to nearly 1000 K [SR2]. The resulting compatibility relations for zone center phonons indicate that transitioning to the orthorhombic phase is associated with the two T_{2g} modes breaking their degeneracy and splitting into three modes [SR3].

The scattering geometries presented in this work were chosen to examine individual modes, with the A_{1g} mode present in the parallel, and the two T_{2g} modes present in the crossed. The $T_{2g}(2)$ mode occurs at ≈ 65 cm^{-1} and represents antiphase vibration between the *A*-site (Pb) and the O_6 octahedra, the $T_{2g}(1)$ mode occurs at ≈ 389 cm^{-1} represents antiphase vibration between the *B*-site (Mg, W) and the O_6 octahedra, and the A_{1g} mode occurs at ≈840 cm^{-1} and represents *B*-O-*B*' (Mg-O-W) stretching via antiphase O_6 octahedral breathing modes. Thus, the $T_{2g}(2)$ mode corresponds to Pb ordering and thus polar ordering, whereas the A_{1g} mode represents the O_6 octahedra dynamics and its distorted variants.

The cubic T_{2g} mode at ≈ 65 cm^{-1} begins to break into multiple lines at around 200 K in the below-T_C orthorhombic phase. These resulting modes change slowly between 200 K and 100 K during the intermediate orthorhombic phase. Below 100 K, the structure appears to settle into what has been previously called the "orthorhombic antiferroelectric" phase [SR2], where the predominant remnant of the T_{2g} mode has stabilized at a higher energy of ≈ 80 cm^{-1}, and at the lowest temperature of 10 K another low energy mode is enhanced. Such temperature dependent changes and transitions can also be seen in the cubic A_{1g} mode at ≈ 840 cm^{-1}. In the intermediate orthorhombic phase, we identify the main mode at that same energy in addition to two higher energy modes, denoted by circles, that we attribute to locally distorted regions. Upon reaching the ≈100 K threshold, the intensity of the A_{1g} mode increases in intensity and decreases its linewidth, indicating longer range ordering of the parent phase. The onset of a previously reported mode ≈ 720 cm^{-1}, likely due to another variant of distortion, also occurs below 100 K.

Analysis of the hysteresis loops

The origin of the standard double AFE loop is well-understood. As the applied *E*-field is increased, a high enough electric field eventually makes the free energies of the AFE and FE phases equal ($E_{AFE=FE}$); however, the AFE→FE transition occurs at a slightly higher *E* field because due to the need to overcome a kinetic barrier at $E_{AFE=FE}$ in the first-order phase transition between the AFE and FE phases. Then, when the applied field is decreases, the FE phase is kinetically trapped at $E_{AFE=FE}$ and the FE→AFE transition occurs at a field slightly lower than $E_{AFE=FE}$. As the temperature decreases, the magnitude of the kinetic barrier at $E_{AFE=FE}$ relative to the temperature increases as well, necessitating a larger difference between $E_{AFE\rightarrow FE}$ ($E_{FE\rightarrow FE}$) and $E_{AFE=FE}$ to drive the AFE→FE (FE→AFE) phase transition. At the first approximation, $E_{AFE=FE}$ can be assumed to be the average of $E_{AFE\rightarrow FE}$ and $E_{FE\rightarrow AFE}$.

In the hysteresis loop, the switching of the FE phase to the AFE phase and vice versa leads to larger *P* changes and therefore a high slope of *P* versus *E* field. By contrast, when the material is fully in the FE phase, the increasing *P* is only due to the increase in the intrinsic *P* of the FE phase and the hysteresis loop shows slow variation with *E*-field. Similarly, when a material is fully in the AFE phase, the applied *E*-field changes the intrinsic *P* of the AFE from zero, leading to slow changes in *P* with variation in the applied *E*-field. When a rapid change in *P* is observed in a hysteresis loop (*e.g.* from high *P* values to low *P* values), this indicates that the system is free to switch from the FE phase to the AFE phase for the double loop or to the FE phase with a different polarization in the FE hysteresis loop because it is no longer trapped kinetically in the original FE state. In this case the switching proceeds to the thermodynamically favored state. Therefore, when we observe a change from the slow *P* changes to fast *P* changes and then again to slow *P* changes at negative *E*-field for T < 60 K, this indicates that the system is free to change from the *P* phase induced by the positive applied E field to the thermodynamically preferred state at negative E fields. However, the fact that the P change after rapid switching is slow with P close to zero indicates that the thermodynamically preferred state contains the AFE phase. If the FE phase was thermodynamically preferred, the switching would proceed from high *P* to low *P* directly as in the standard FE hysteresis loops, bypassing the intermediate *P* state observed for our $PbMg_{0.5}W_{0.5}O_3$ films for T < 60 K. Therefore, the thermodynamically preferred state at these temperatures is a mixture of the FE and AFE phases.

Compressive strain induced by the changes in the lattice parameter of the $NbScO_3$ substrate at low temperatures

The bulk $PbMg_{0.5}W_{0.5}O_3$ exhibits almost no volume change (thermal expansion coefficient is close to zero) at low temperatures [SR4]. On the other hand, the thermal expansion coefficient of bulk $NbScO_3$ (lattice constant a=5.575 Å, b=5.776 Å, c=8.003 Å at room temperature) has been reported [SR5], as shown in Table S1. Based on the equation:

$$a(T) = a(T_0) \cdot [1 - \alpha \cdot (T_0 - T)]$$

where *a* (*T*) is lattice constant at the specific temperature, T_0=293 K, the lattice constant of $NbScO_3$ at low temperatures could be estimated. Additionally, the $PbMg_{0.5}W_{0.5}O_3$ films are fully epitaxial grown on $NbScO_3$ without relaxation (Fig. 1e). Hence, compressive strain induced by the changes in the lattice parameter of the $NbScO_3$ substrate at 10 K is ~ 0.3 %, leading to the phase transition from AFE to FE phase upon cooling.

Table S1 thermal expansion coefficient of $NdScO_3$.

	$\alpha_{11}(10^{-6}K^{-1})$	$\alpha_{22}(10^{-6}K^{-1})$	$\alpha_{33}(10^{-6}K^{-1})$	$\alpha_{\text{average}}(10^{-6}K^{-1})$
$NdScO_3$	12.9	4.2	9.3	8.8

Phase Stabilization and Design Rules

Because the interplay and relative energies between the FE and AFE phase play such a crucial role in the macroscopic dielectric properties of bulk $PbMg_{0.5}W_{0.5}O_3$, here we propose some simple design rules for

how these states can be promoted in the bulk crystal. Additionally, the formation of any kind of AFE domain walls in the material will be unfavorable relative to the FE state. Therefore, existing domain walls created during growth or field cycling will be guided thermodynamically to combine to form either the AFE ground state or the stable FE state, particularly at low temperatures.

We posit that the relative phase energies can be controlled by modulating the cell volume. This can be done by manipulating strain of the material with substrates, or though chemical pressure effects by doping $PbMg_{0.5}W_{0.5}O_3$ with smaller ions. $PbMg_{0.5}W_{0.5}O_3$ is already unique among Pb-based perovskites though its complicated phase diagram and atomic configurations, and modifications to enhance this feature can serve both to achieve enhanced electrochemical properties and to enable new functionalities with designer AFE/FE transitions. Creative design of the relative phase stability can also enable the observed relaxor behavior to be observed at higher temperatures, making the material potentially more relevant for implementation in existing device schemes.

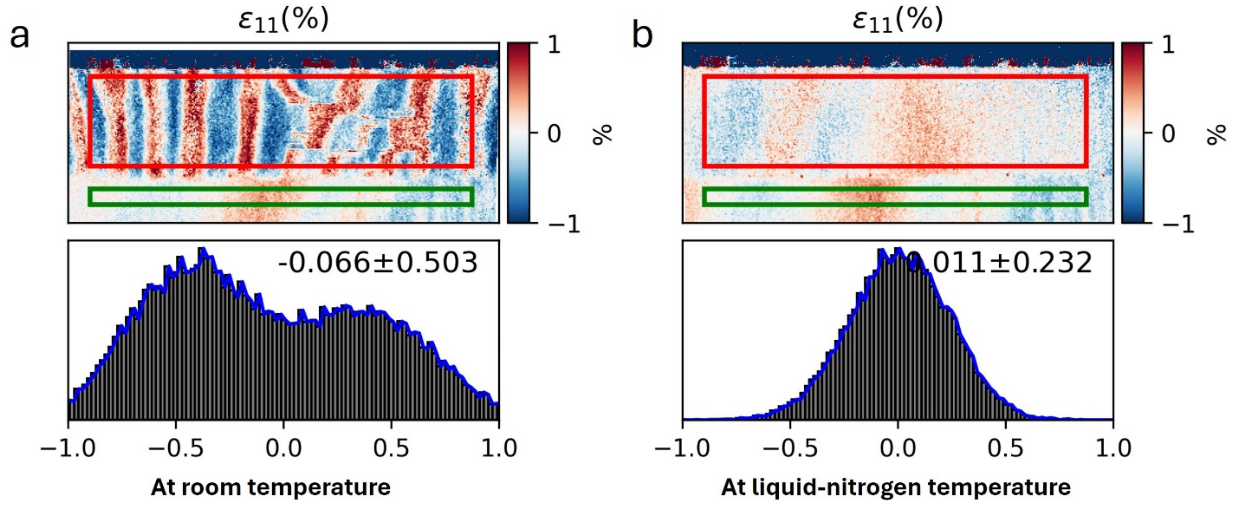

Fig. S5. **Film substrate mismatch from STEM-NBED.** The lattice mismatch between the film (red box region) with respect to the average of the substrate (green box region) at **a,** room temperature and **b,** liquid-nitrogen temperature.

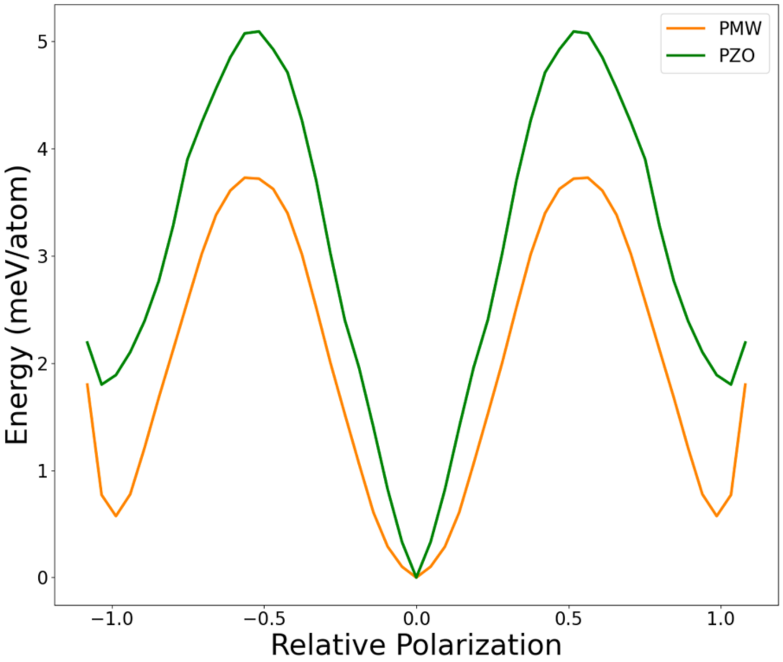

Fig. S6 DFT-generated triple well potential for both $PbMg_{0.5}W_{0.5}O_3$ and $PbZrO_3$. In $PbMg_{0.5}W_{0.5}O_3$, individual dipoles only must rotate 90 degrees to switch phases, resulting in a more stable FE phase and a much lower switching barrier.

References

SR1. M. Liegeois-Duyckaerts and P. Tarte, "Vibrational studies of molybdates, tungstates and related compounds-III. Ordered cubic perovskites $A_2B(II)B(VI)O_6$," Spectrochimica Acta 30A, 1771-1786 (1974).

SR2. A. Kania, E. Jahfel, G. E. Kugel, K. Roleder and M. Hafid, A Raman Investigation of the ordered complex perovskite $PbMg_{0.5}W_{0.5}O_3$, J. Phys.: Condens. Matter. 8, 4441-4453 (1996).

SR3. G. Baldinozzi, P. Sciau and A. Bulou, "Raman study of the structural phase transition in the ordered perovskite Pb_2MgWO_6," J. Phys.: Condens. Matter. 7, 8109-8117 (1995).

SR4. Baldinozm, G., Sciau, P. & Buffat, P.-A. Investigation of the Orthorhombic Structures of Pb_2MgWO_6 and Pb_2CoWO_6. Solid State Communications 86, 541-544 (1993).

SR5. Uecker, R. et al. Properties of rare-earth scandate single crystals (Re=Nd−Dy). Journal of Crystal Growth 310, 2649-2658 (2008).
