# Supplementary material for: Relaxor behavior in rocksalt cation-ordered material induced by (anti)ferroelectric phase competition
Source: Nat Commun. 2026 Jun 23;17:7566. doi: 10.1038/s41467-026-74237-z (PMC13416127; doi:10.1038/s41467-026-74237-z)
Supplement: Supplementary file 2 — Transparent Peer Review file [file 41467_2026_74237_MOESM2_ESM.pdf]

nature portfolio

Peer Review File

Relaxor behavior in rocksalt cation-ordered material induced by (anti)ferroelectric phase competition

Corresponding Author: Professor Jonathan Spanier

This file contains all reviewer reports in order by version, followed by all author rebuttals in order by version.

Version 0:

Reviewer comments:

Reviewer #1

(Remarks to the Author)
The authors reported relaxor behavior in fully cation-ordered antiferroelectric PbMg0.5W0.5O3 epitaxial films, which originates from spontaneous low-temperature phase competition. By combining various characterization techniques with theoretical calculations, they elucidate the structure and energetics of these films, confirming their long-range rocksalt B-cation order. The study proposes a novel approach to regulating the performance of relaxor materials via engineered phase competition. The following comments are provided.
(1) The manuscript states that 100-nm-thick PbMg0.5W0.5O3 films were grown on NdScO3 (110) substrates, but the rationale for this specific substrate choice is not explained. Substrate selection is critical for epitaxial growth, as it directly influences film crystallinity and interfacial properties.
(2) From Figure 2, it can be seen that the HAADF-STEM image of the PbMg0.5W0.5O3 film interface at 298 K was taken along the pseudocubic [110] axis, while the Cryo-STEM image at 103 K was taken along the pseudocubic [100] axis. Therefore, why not compare them under the same zone axis? Providing additional HAADF-STEM images at 298 K along the pseudocubic [100] axis could be useful for comparison. This is more relevant to studying temperature-induced changes in the AFE structure.
(3) Figure 3f plots the temperature dependence of Pr, PFE,0, and Pmixed. However, the P-E loops in Fig. 3a and c were obtained using different electric fields at various temperatures. This inconsistency may affect the accuracy of the extracted parameters (Pr, PFE,0, and Pmixed).
(4) To fully assess the quality of the deposited films, it is necessary to characterize their surface morphology. Please provide AFM or SEM images for this purpose. Additionally, the method used to determine the film thickness should be clearly stated.
(5) The clarity of some images, such as Fig. S3, should be improved. Please replace them with higher-resolution versions to ensure that all presented information is easily accessible to readers.

Reviewer #2

(Remarks to the Author)
This study on the relaxor ferroelectric $PbMg_{0.5}W_{0.5}O_3$ reveals a novel mechanism for generating relaxor behavior, which is scientifically significant and innovative (e.g., it is the first to demonstrate that relaxor behavior can also be induced through ferroelectric/antiferroelectric phase competition in a fully cation-ordered material). However, when examined from a purely technical or experimental perspective, the article itself mentions or even implies some potential shortcomings or unresolved issues.
1.The article attributes the relaxor behavior entirely to AFE/FE phase competition and suggests that the chemical disorder required by traditional relaxors is unnecessary. However, it cannot be completely ruled out that other factors (such as possible residual defects in the film, interface effects, oxygen vacancies, and other charged defects) may contribute to the dielectric dispersion, especially given the limited measurement frequency range.
2. Regarding the measurement voltage used for dielectric relaxation, is this energy sufficient to drive the mutual transformation between the ferroelectric and antiferroelectric phases, thereby causing the dielectric relaxation?
3. Relying on epitaxial strain as a means to stabilize the FE phase introduces uncertainties in the actual strain state and the calculations of temperature-dependent lattice contraction (especially since partial relaxation may occur at the interface). This could affect the precise estimation of the FE phase energy levels and the peak of the relaxor behavior.

Reviewer #3

(Remarks to the Author)
The manuscript “Relaxor behavior in rocksalt cation-ordered material induced by (anti)ferroelectric phase competition” by Jonathan E. Spanier et al. reports relaxor behaviour originating from the competition between ferroelectric and antiferroelectric phases in a fully cation-ordered antiferroelectric PbMg0.5W0.5O3.

Comments:
Explain/comment on the crystal structure in the temperature range where the dispersion is observed? Does it remain Pnma at long ranges, or—if ferroelectric (FE) and antiferroelectric (AFE) phases coexist—which structure corresponds to the FE phase? Does this FE-like behaviour in the hysteresis loop come from the local ordering? If yes, comment/explain the structure/atomic ordering at local level.
Complementing various techniques viz.,
i) Pair distribution functon(obtained from Fourier transform of the diffraction data of sintered powders) analysis,
ii) Raman analysis,
iii) and X-ray diffraction data analysis, in conjunction with physical properties like temperature-dependent dielectric and FE-loop analysis, can reveal/give an idea of the structure at long- and short ranges thereby revealing the underlying science of the material.
The major claim of the authors is that the relaxor behaviour is due to the coexistence of ferroelectric and antiferroelectric phases.
A scientific model which evolves relaxor like behaviour from the competition between ferroelectric (FE) and antiferroelectric (AFE) phases is recommended.
Such demonstrations (relaxor like behaviour originating from the competition between ferroelectric (FE) and antiferroelectric (AFE) phases) can be seen in the earlier reports: for e.g. pure NaNbO3, where the antiferroelectric (SG: Pbcm) to ferroelectric (SG: R3c) phase transition starts at T~280 K (as it is accompanied by the coexistence of FE and AFE phases within the temperature range 15 K ≤ T ≤ 280 K) and is associated with diffuse dielectric behaviour exhibiting relaxor-like characteristics [1-2].
Moreover, authors have reported relaxor behaviour in Na0.5Bi0.5TiO3 (NBT) (where the dielectric curve exhibits frequency dependence at low temperatures, T ~ 200°C), corresponding to an antiferroelectric-to-ferroelectric phase transition [3-5].
Usually, relaxor behaviour results from short-range order that may arise from size/charge differences, defects, etc. How will the authors ascertain that the relaxor behaviour is not due to cross-site (B/B’ site) disorder or any kind of defects?
Raman Spectroscopy:
How is the intermediate orthorhombic phase between 100 and 200 K different from Pnma?
Authors report an orthorhombic antiferroelectric phase below T ~ 100 K. Explain how it is different from the high temperature phases (crystal structures). How do you link it with AFE-FE transition?
Deconvoluting the peaks in the Raman spectra and plotting their FWHM as a function of temperature may help in fixing the critical temperatures and throw light on various phase transitions in the materials at atomic level.
In conclusion, the presented results do not introduce any novel scientific findings nor do they advance the existing understanding in this area of research. The authors’ primary claim—that “these findings demonstrate a new paradigm for designing relaxor material properties through engineered phase competition”—has already been reported in earlier studies where similar phenomena have been observed in pure $NaNbO_3$ and $Na_{1/2}Bi_{1/2}TiO_3$ systems, where the antiferroelectric-to-ferroelectric phase transition is accompanied by diffuse dielectric behaviour exhibiting relaxor-like characteristics.

In addition, many of the explanations provided in the manuscript would benefit from further elaboration, additional experiments, and a more detailed analysis of the experimental results. Improved integration of observations from different experimental techniques may help and clarify the scientific context. Accordingly, I do not recommend the manuscript for publication in Nature Communications in its present form.
References:
1. Mishra, S. K., Mittal, R., Pomjakushin, V. Y., & Chaplot, S. L. (2011). Phase stability and structural temperature dependence in sodium niobate: a high-resolution powder neutron diffraction study. Physical Review B—Condensed Matter and Materials Physics, 83(13), 134105.
2. Lanfredi, S., Lente, M. H., & Eiras, J. A. (2002). Phase transition at low temperature in NaNbO3 ceramic. Applied physics letters, 80(15), 2731-2733.
3. Dorcet, V., Trolliard, G., & Boullay, P. (2008). Reinvestigation of phase transitions in Na0. 5Bi0. 5TiO3 by TEM. Part I: First order rhombohedral to orthorhombic phase transition. Chemistry of Materials, 20(15), 5061-5073.
4. Isupov, V. A. (2005). Ferroelectric Na0. 5Bi0. 5TiO3 and K0.5Bi0.5TiO3 perovskites and their solid solutions. Ferroelectrics, 315(1), 123-147.
5. Sakata, K., & Masuda, Y. (1974). Ferroelectric and antiferroelectric properties of (Na0.5Bi0.5)TiO3-SrTiO3 solid solution ceramics. Ferroelectrics, 7(1), 347-349.

Version 1:

Reviewer comments:

Reviewer #1

(Remarks to the Author)
The authors have thoroughly revised the manuscript in response to the reviewers′ comments. All previously raised issues have been resolved. I therefore recommend the manuscript for acceptance.

Reviewer #2

(Remarks to the Author)
The authors respond my comments well, it can be accepted as it is.

Reviewer #3

(Remarks to the Author)
The authors have made significant effort to revise the manuscript and incorporate the suggested changes, though the manuscript can still be improved with further clarification and better presentation of the results.
The authors state that PbMg0.5W0.5O3 is fully cation-ordered compared to Na0.5Bi0.5TiO3, while both materials still exhibit cation disorder at the B- and A-sites, respectively. Therefore, if the relaxor behaviour in NBT is attributed to cation disorder, a similar interpretation may also apply to PMW.
In the deconvolution of the A1g mode (~840 cm-1), the plotted Raman shift shows several anomalies. What do these anomalies correspond to?
The FWHM is expected to increase with temperature, but an anomaly is observed around 300 K that requires further explanation.

Version 2:

Reviewer comments:

Reviewer #3

(Remarks to the Author)
I recommend the manuscript for acceptance.

Open Access This Peer Review File is licensed under a Creative Commons Attribution 4.0 International License, which permits use, sharing, adaptation, distribution and reproduction in any medium or format, as long as you give appropriate credit to the original author(s) and the source, provide a link to the Creative Commons license, and indicate if changes were made.
In cases where reviewers are anonymous, credit should be given to 'Anonymous Referee' and the source.
The images or other third party material in this Peer Review File are included in the article’s Creative Commons license, unless indicated otherwise in a credit line to the material. If material is not included in the article’s Creative Commons license and your intended use is not permitted by statutory regulation or exceeds the permitted use, you will need to obtain

permission directly from the copyright holder.
To view a copy of this license, visit https://creativecommons.org/licenses/by/4.0/

REVIEWER COMMENTS

Reviewer #1 (Remarks to the Author):

The authors reported relaxor behavior in fully cation-ordered antiferroelectric $PbMg_{0.5}W_{0.5}O_3$ epitaxial films, which originates from spontaneous low-temperature phase competition. By combining various characterization techniques with theoretical calculations, they elucidate the structure and energetics of these films, confirming their long-range rocksalt B-cation order. The study proposes a novel approach to regulating the performance of relaxor materials via engineered phase competition. The following comments are provided.

(1) The manuscript states that 100-nm-thick $PbMg_{0.5}W_{0.5}O_3$ films were grown on $NdScO_3$ (110) substrates, but the rationale for this specific substrate choice is not explained. Substrate selection is critical for epitaxial growth, as it directly influences film crystallinity and interfacial properties.

Response: We thank the reviewer for the valuable comments. Lattice matching between films and substrates is a critical parameter for epitaxial film growth. We chose the $NdScO_3$ (NSO) (110) substrate for $PbMg_{0.5}W_{0.5}O_3$ film deposition because it yields the smallest lattice mismatch, which helps to reveal the intrinsic properties of $PbMg_{0.5}W_{0.5}O_3$ with negligible or minimal strain at room temperature. The corresponding lattice constant values are shown below. We have added the phrase “with an excellent lattice matching” marked by yellow shadows in the last paragraph on page 2.

Pseudocubic structure: PMW a= 3.972 Å, b=4.0268 Å, c= 4.0268 Å;

NSO (110), a=4.0014, b=4.002

(2) From Figure 2, it can be seen that the HAADF-STEM image of the $PbMg_{0.5}W_{0.5}O_3$ film interface at 298 K was taken along the pseudocubic [110] axis, while the Cryo-STEM image at 103 K was taken along the pseudocubic [100] axis. Therefore, why not compare them under the same zone axis? Providing additional HAADF-STEM images at 298 K along the pseudocubic [100] axis could be useful for comparison. This is more relevant to studying temperature-induced changes in the AFE structure.

Response: We thank the reviewer for the constructive suggestions. Room-temperature STEM images acquired along the pseudocubic [100] axis are presented below (Fig. R1). The pseudocubic [110] orientation is more appropriate for illustrating the crystal structure and superlattice reflections, whereas the pseudocubic [100] orientation is employed to visualize the distribution of polarization directions. We have added room-temperature STEM along [100] axis in the Supplementary Information.

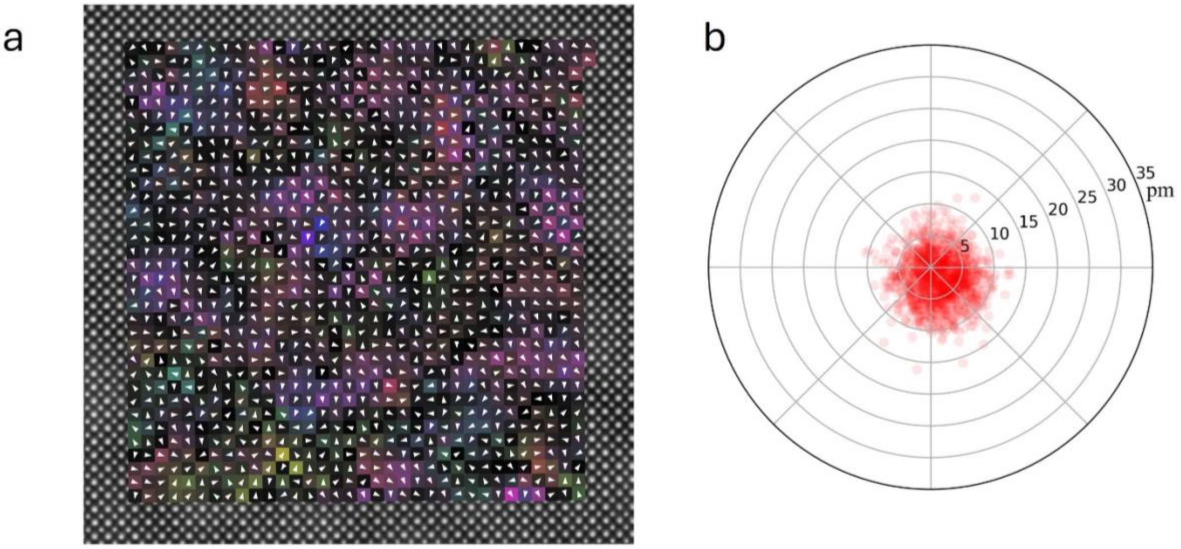

Fig. R1 a. STEM image at room temperature along the pseudocubic [100]-type projection with disordered cation displacements. b. Distribution of cation displacements.

(3) Figure 3f plots the temperature dependence of Pr, PFE,0, and Pmixed. However, the P-E loops in Fig. 3a and c were obtained using different electric fields at various temperatures. This inconsistency may affect the accuracy of the extracted parameters (Pr, P_{FE},0, and Pmixed).

Response: We appreciate the reviewer's comment. Ideally, a larger applied electric field is preferable; however, in practical devices, factors such as the material's dielectric breakdown field must be considered. An excessively high electric field may cause irreversible damage, making it difficult to use a uniform electric field to characterize polarization switching across different temperatures. As shown in Fig. 3c, the electric field required to achieve polarization saturation in the $PbMg_{0.5}W_{0.5}O_3$ films varies with temperature. Our criterion for the applied electric field is to ensure complete polarization switching, with a similar additional electric field superimposed. Although the applied electric fields differ, the hysteresis loops corresponding to the ascending and descending electric field ramps nearly overlap near the maximum field. Thus, this effect is minor and negligible.

(4) To fully assess the quality of the deposited films, it is necessary to characterize their surface morphology. Please provide AFM or SEM images for this purpose. Additionally, the method used to determine the film thickness should be clearly stated.

Response: We thank the reviewer for the suggestions. We have added AFM results to show the surface morphology in the supplementary material, as shown in Fig. R2. We have also stated that the Laue oscillations of the $PbMg_{0.5}W_{0.5}O_3$ peak were used to determine the film thickness in the revised manuscript on page 3.

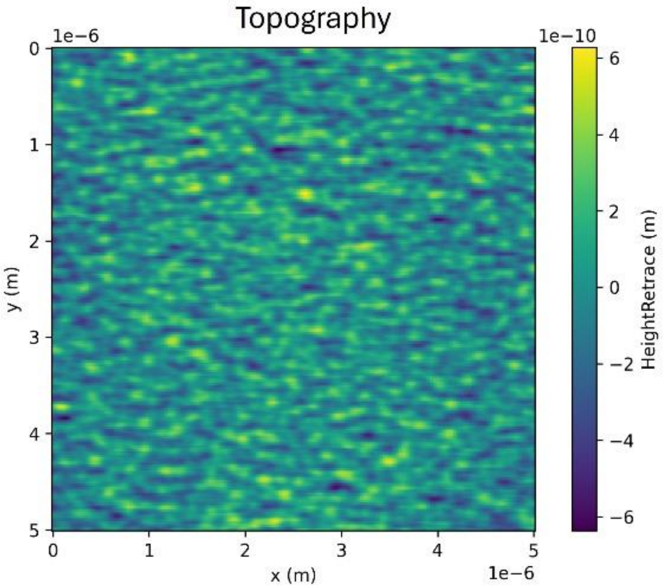

Fig. R2. AFM image for 100-nm-thick $PbMg_{0.5}W_{0.5}O_3$ films with an area of 5μm×5μm.

(5) The clarity of some images, such as Fig. S3, should be improved. Please replace them with higher-resolution versions to ensure that all presented information is easily accessible to readers.

Response: Thanks for your suggestions, we have replaced Fig. S3 (now it is Fig. S5 in the revised manuscript) with a higher-resolution version.

We thank the reviewer again for the helpful assistance. We believe all the issues raised have been fully addressed.

Reviewer #2 (Remarks to the Author):

This study on the relaxor ferroelectric $PbMg_{0.5}W_{0.5}O_3$ reveals a novel mechanism for generating relaxor behavior, which is scientifically significant and innovative (e.g., it is the first to demonstrate that relaxor behavior can also be induced through ferroelectric/antiferroelectric phase competition in a fully cation-ordered material). However, when examined from a purely technical or experimental perspective, the article itself mentions or even implies some potential shortcomings or unresolved issues.

Response: We greatly appreciate the reviewer for the positive evaluation and kind recognition of the scientific significance and innovation of our work on the relaxor ferroelectric $PbMg_{0.5}W_{0.5}O_3$. We carefully address each of the raised concerns in the following:

1.The article attributes the relaxor behavior entirely to AFE/FE phase competition and suggests that the chemical disorder required by traditional relaxors is unnecessary. However, it cannot be completely ruled out that other factors (such as possible residual defects in the film, interface effects, oxygen vacancies, and other charged defects) may contribute to the dielectric dispersion, especially given the limited measurement frequency range.

Response: This is an excellent question. We acknowledge that no perfect crystal exists, and defects are inevitable in all materials. Relaxor behavior can be caused by defects, as for example explored in (*Appl. Phys. Lett.* 87, 192905 (2005), https://doi.org/10.1063/1.2125110 and *Phys. Rev. Materials* **5**, 064415 (2021), DOI: https://doi.org/10.1103/PhysRevMaterials.5.064415). However, to induce relaxor behavior, a fairly high defect concentration is required. While defects and especially oxygen vacancies are always present in perovskite crystals, ceramics and films, the normal content of oxygen vacancies does not give rise to relaxor behavior in AFE film and bulk systems such as $PbZrO_3$ (PZ) and $PbHfO_3$ (PH) and $PbMg_{0.5}W0_{0.5}O_3$ (PMW), and a high concentration of O vacancies and defects usually gives rise to high leakage and asymmetric hysteresis loops. In this work, we grew high-quality single-crystal films of PMW that do not display any of the symptoms of high defect content and are similar to the high-quality PZ and PH films. Therefore, they are unlikely to have more than the standard concentration of O vacancies and other defects. Such a low concentration was not observed to give rise to relaxor behavior in the previously studied PZ and PH films. Therefore, the relaxor behavior observed in our work is highly unlikely to be due to defects.

To address the referee's comment, we have added a discussion about the influence of defects in the revised manuscript. "*Defects such as oxygen vacancies often have complex effects on conventional ferroelectrics and in some cases their presence at high concentration gives rise to relaxor behavior as well as high leakage and asymmetric hysteresis loops (Appl. Phys. Lett.* 87, 192905 (2005), and *Phys. Rev. Materials* **5**, 064415 (2021)) *In this work, to reduce the effects of defects, high-quality epitaxial films were obtained by optimizing growth conditions followed by post-annealing in a high-oxygen atmosphere. These films exhibit low leakage and symmetric hysteresis loops, indicating a low concentration of defects such as oxygen vacancies in the films. Such low concentrations of defects do not give rise to relaxor behavior in films of AFE materials such as $PbZrO_3$. Therefore, it is unlikely that the observed relaxor behavior is due to the presence of oxygen vacancies and other defects, and rather arises from the competition between the AFE and FE phase as discussed above.*"

2. Regarding the measurement voltage used for dielectric relaxation, is this energy sufficient to drive the mutual transformation between the ferroelectric and antiferroelectric phases, thereby causing the dielectric relaxation?

Response: We appreciate the reviewer for raising this important question. The voltage used to measure the dielectric response is quite small, raising the question whether it can lead to the switching between FE and AFE phases. To answer this questions, it is useful to consider the dielectric response and dynamics in classic relaxors such as PMN. In classic relaxors, the small applied voltage does not lead to full switching of the small polar nanodomains but rather changes the orientation of the dipoles at the boundaries between these nanodomains where the energy landscape for dipole rotation is very shallow. Similarly, we believe that the coexistence of the FE and AFE phases in small domains leads to the formation of a large number of boundaries between the FE and AFE phases. At these boundaries, the energy landscape for the rotation of individual dipoles generated by the Pb off-center displacement is quite shallow with very small barrier to dipole rotations and therefore the dipoles will respond in a kinetics-limited manner even to a small applied voltage, which will give rise to dielectric relaxation. As the AFE or FE phase becomes dominant at high or very low temperature, respectively, the barrier for P rotation at the boundaries between the FE and AFE phase regions become quite large and the relaxation disappears. This picture is exactly analogous to the understanding of the origin of the dielectric relaxation in classic relaxor system; however, in classic relaxors the boundaries are between domains with slightly different orientation of local polarization whereas in our system the domain boundaries are between FE and AFE phases.

3. Relying on epitaxial strain as a means to stabilize the FE phase introduces uncertainties in the actual strain state and the calculations of temperature-dependent lattice contraction (especially since partial relaxation may occur at the interface). This could affect the precise estimation of the FE phase energy levels and the peak of the relaxor behavior.

Response: We appreciate the reviewer's constructive comments. Regarding the reviewer's concern about strain relaxation at low temperatures:

Based on reciprocal space mapping (RSM) and other structural characterizations (Fig. 1 and 2), we confirm that the $PbMg_{0.5}W_{0.5}O_3$ thin film is fully epitaxially grown on NSO substrate at room temperature, with a sharp and continuous interface and no sign of stress relaxation. Upon cooling, owing to the difference in thermal expansion coefficients between the PMW film and the NSO substrate, our calculations indicate that the in-plane compressive strain exerted on the PMW film is merely 0.3%, making strain relaxation highly unlikely.

More importantly, we employed transmission electron microscopy (TEM) to investigate the strain state of the film at both room and low temperatures, and no stress relaxation was observed under either condition, as illustrated in Fig. R3. The parameter ε_{11} represents the in-plane lattice mismatch between the film and the substrate. A lattice mismatch of 0.066% at low temperature confirms the complete absence of stress relaxation. The difference in lattice mismatch between room temperature and liquid-nitrogen temperature lies within the experimental error and can thus be neglected.

In summary, no stress relaxation takes place in the film at low temperatures, and our estimations of the strain variation and the energy evolution of the ferroelectric phase derived from thermal expansion effects are reliable. We have incorporated the TEM-based strain analysis data into the Supplementary Materials.

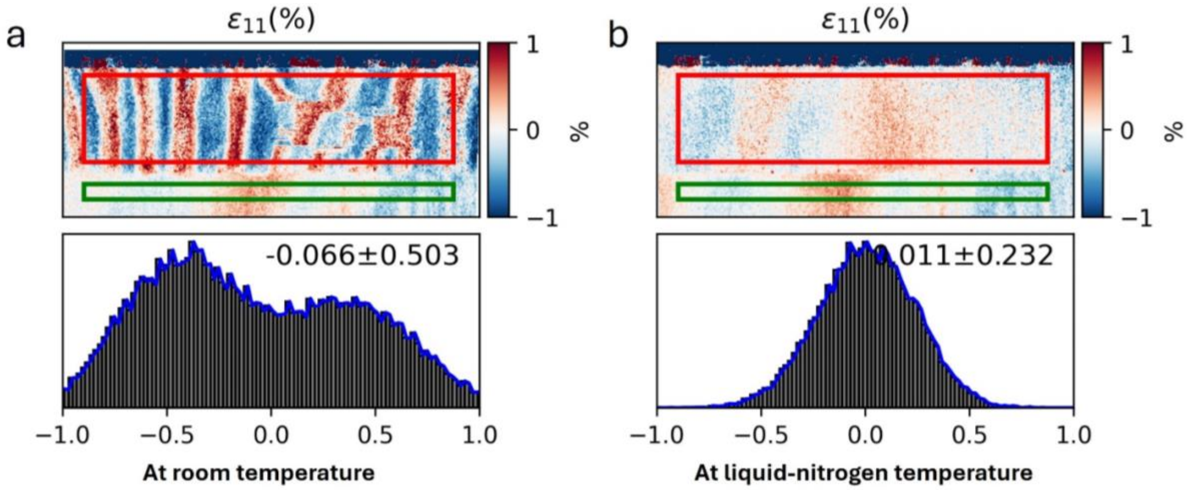

Fig. R3 Strain mapping for $PbMg_{0.5}W_{0.5}O_3$/NSO films using Nano-beam electron diffraction (NBED) four-dimensional STEM. The lattice mismatch between the film (red box region) and the average of the substrate (green box region) **a**, at room temperature and **b**, liquid-nitrogen temperature.

We thank the reviewer again for these valuable suggestions, which have greatly helped us improve the quality of the manuscript.

Reviewer #3 (Remarks to the Author):

The manuscript "Relaxor behavior in rocksalt cation-ordered material induced by (anti)ferroelectric phase competition" by Jonathan E. Spanier et al. reports relaxor behaviour originating from the competition between ferroelectric and antiferroelectric phases in a fully cation-ordered antiferroelectric $PbMg_{0.5}W_{0.5}O_3$.

Comments:
1. Explain/comment on the crystal structure in the temperature range where the dispersion is observed? Does it remain Pnma at long ranges, or—if ferroelectric (FE) and antiferroelectric (AFE) phases coexist—which structure corresponds to the FE phase? Does this FE-like behaviour in the hysteresis loop come from the local ordering?

Response: We thank the reviewer for the comments. Yes, the FE and AFE coexist in the temperature range where the dispersion is observed. The antiferroelectric (AFE) phase adopts the nonpolar *Pnma* (or *Pmcn*) space group. When the antiparallel dipoles align into parallel ferroelectric order, the symmetry is reduced to the polar orthorhombic space group *Amm2*, which is a polar subgroup of Pnma while maintaining a similar structural framework. We believe that the observed ferroelectric-like hysteresis loop originates from local short-range ferroelectric ordering, while the long-range crystal structure retains the nonpolar *Pnma* space group. Such local polar ordering is responsible for the FE-like dielectric and hysteretic behavior in the system. Notably, as the temperature decreases further, the ferroelectric (FE) phase gradually stabilizes and eventually becomes long-range ordered.

2. If yes, comment/explain the structure/atomic ordering at local level. Complementing various techniques viz., i) Pair distribution functon (obtained from Fourier transform of the diffraction data of sintered powders) analysis, ii) Raman analysis, iii) and X-ray diffraction data analysis, in conjunction with physical properties like temperature-dependent dielectric and FE- loop analysis, can reveal/give an idea of the structure at long- and short ranges thereby revealing the underlying science of the material.

Response: We thank the reviewer for the comments. The Raman scattering results provide clear evidence for local distortion and local polar ordering at low temperatures. A new mode appears at ≈720 cm^{-1} below 100 K, consistent with another type of local distortion. Raman observations confirm the existence of local symmetry breaking and local polar regions at low temperatures, while the long-range average structure retains the orthorhombic *Pmcn* (*Pnma*) phase.

On the other hand, the temperature evolution of the hysteresis loops demonstrates that the film transforms from the high-temperature antiferroelectric (AFE) phase to the low-temperature ferroelectric (FE) phase with decreasing temperature. A coexistence or transition region between the FE and AFE phases exists, and this temperature window coincides exactly with the range where dielectric relaxation is observed. This indicates that the relaxation behavior is correlated with the AFE-FE phase transition, and the two phases share a similar structural framework.

We have added sentences on the page 6:

The major claim of the authors is that the relaxor behaviour is due to the coexistence of ferroelectric and antiferroelectric phases. A scientific model which evolves relaxor like behaviour from the competition between ferroelectric (FE) and antiferroelectric (AFE) phases is recommended.

Response: We agree with the referee that a quantitative model which shows how relaxor-like behavior can arise from FE-AFE competition would be very useful for our study of relaxation in PMW films. Unfortunately, there is currently no widely accepted simple model that gives rise to relaxor dielectric relaxation is available even for classic relaxor compounds.

Models of relaxor behavior can be divided into three categories based on their level of abstraction. The first is an analytical theory that shows how the key features of relaxors such as dielectric relaxation and diffuse phase transition appear due to chemical disorder while showing quantitative agreement with experimental results and being able to predict Vogel-Fulcher behavior and parameters such as the diffusion parameter γ and the dispersion parameter $\Delta T_{\varepsilon,\max}$. No such theory is currently available. While the work by Pirc and Blinc in 2007 (*Phys. Rev. B* **76**, 020101(R) (2007), DOI: https://doi.org/10.1103/PhysRevB.76.020101) suggested how the Vogel-Fulcher behavior can arise from the model of percolating polar nanoregions, even that model does not fully address the experimental dielectric relaxation results, and furthermore is based on the assumption of PNR in non-polar matrix which has been shown to be incorrect theoretically and experimentally.

At the second level, Ising-type models can be used to model relaxor behavior where each unit cell is considered to be a spin (or a dipole) that interacts with its neighbors through several simple interactions. Multiple studies in the literature have used this approach but no consensus has been reached regarding the correct simple model and none of the models correctly reproduce all of the features of relaxor behavior. To large extent, these models can be adjusted to reproduce the desired results and thus are of limited utility for verifying that FE-AFE coexistence can give rise to relaxor behavior.

At the third level, relaxors can be studied using atomistic potentials and effective Hamiltonians parameterized from DFT which reproduce the energy landscape of DFT calculations and allow molecular dynamics simulations on much larger scales. These methods have successfully reproduced the dielectric relaxation observed experimentally, but do not in and of themselves provide much insight without extensive analysis of dynamics and structure obtained in the simulations. While these methods in principle can be used to simulate the FE-AFE coexistence in PMW films, such investigation would require a very large research effort involving construction and validation of potentials and MD simulations at very large length- and timescales and would constitute a significant stand-alone study. Thus, such MD simulations are outside the scope of this work.

To address the referee's comments, we have added a paragraph discussing the lack of available simple models for relaxor behavior and pointing out the need to conduct large-scale MD simulations in future work to confirm that FE-AFE coexistence gives rise to relaxor behavior observed in our PMW films.

"*Further insights into the connection between the AFE-FE phase competition and observed relaxor behavior would benefit from theoretical modeling of relaxor dynamics in PMW. However, simple models for understanding relaxor dynamics are currently lacking while MD simulations of coexisting AFE and FE phases in PMW would are highly non-trivial and require parameterization and validation of atomistic potentials as well as simulations at large length- and timescales. Such simulations are outside the scope of this work and will be conducted in future research*."

Such demonstrations (relaxor like behaviour originating from the competition between ferroelectric (FE) and antiferroelectric (AFE) phases) can be seen in the earlier reports: for e.g. pure $NaNbO_3$, where the antiferroelectric (SG: Pbcm) to ferroelectric (SG: R3c) phase transition

starts at T~280 K (as it is accompanied by the coexistence of FE and AFE phases within the temperature range 15 K ≤ T ≤ 280 K) and is associated with diffuse dielectric behaviour exhibiting relaxor-like characteristics [1-2]. Moreover, authors have reported relaxor behaviour in $Na_{0.5}Bi_{0.5}TiO_3$ (NBT) (where the dielectric curve exhibits frequency dependence at low temperatures, T ~ 200°C), corresponding to an antiferroelectric-to-ferroelectric phase transition [3-5]. Usually, relaxor behaviour results from short-range order that may arise from size/charge differences, defects, etc. How will the authors ascertain that the relaxor behaviour is not due to cross-site (B/B' site) disorder or any kind of defects?

Response: This is an excellent question, and we thank the reviewer for referencing the relevant literature on $NaNbO_3$ and $Na_{0.5}Bi_{0.5}TiO_3$ (NBT) and providing us with an opportunity to clarify the novelty of the results obtained in our work. The studies on NBT cited by the referee (as well as other studies of NBT) do clearly show dispersion in the region of the FE-AFE phase transition. However, this dispersion cannot be unambiguously attributed to the FE-AFE phase competitions because NBT shows cation disorder on the A-site with Na^+ and Bi^{3+} arranged somewhat randomly. Thus, NBT is similar to PMN and other classic relaxors in that it shows a disordered arrangement of heterovalent ions on the perovskite cation sublattice (A-site in the case of NBT, B-site in the case of PMN). Such disordered cation arrangement is known to be the origin of relaxor behavior in PMN and is also likely to be the cause of relaxor behavior in NBT, similar to the disordered arrangement of heterovalent cations on the A-site in $Pb_{1-x}La_xZr_{0.65}Ti_{0.35}O_3$ (PLZT). Furthermore, the dispersion in NBT is observed for a wide range of temperatures beyond the region of the FE-AFE phase transition, suggesting that the origin of relaxor behavior in NBT is not related to FE-AFE competition and rather should be attributed to the cation disorder, similar to classic relaxors such as PMN.

Regarding $NaNbO_3$, the referee is correct that low-temperature dispersion has been observed in $NaNbO_3$ in the region below the AFE-FE phase transition and has been ascribed to the response of domain walls between small domains of meta-stable phases. However, $NaNbO_3$ is well-known to be an extremely complex material that shows no less than 7 phases at different temperatures indicating very small energy differences between the phases which are controlled by subtle interactions, as discussed for example in *Phys. Rev. B* 97, 174106 (2018). This makes $NaNbO_3$ sensitive to the details of sample processing. As stated by Randall and co-workers in their paper on an incommensurate phase in $NaNbO_3$ (*Appl. Phys. Lett.* 107, 112904 (2015) https://doi.org/10.1063/1.4930067), "it should be cautiously pointed out that the phase structure of $NaNbO_3$ is extremely sensitive to various factors. As reported in the literature so far, the structural behavior of $NaNbO_3$ can vary remarkably among the $NaNbO_3$ powders, as-sintered ceramics, single crystals, and thin films. Specifically, the phase state is affected by homogeneity, synthesis route, particle size effect, strain environment, thermal history, electrical treatment conditions, and stoichiometric ratio of the chemicals." For such a sensitive material, low-temperature dispersion may be due to effects of defects such as Na and O vacancies at low concentration that are introduced during sample synthesis and processing. Furthermore, recent studies (Harikrishnan et al, *Nat. Mater.* **24**,1433 (2025), https://doi.org/10.1038/s41563-025-02205-x, Xu et al. *Nat. Commun.* **16**, 5438 (2025), https://doi.org/10.1038/s41467-025-60568-w) have demonstrated that the FE and AFE phases in $NaNbO_3$ originate from complex displacements of oxygen anions which is fundamentally different from the cation-displacement-driven FE and AFE phases in standard FE perovskites (*e.g.* Pb- and Bi-based ABO_3, as well as ABO_3 solid solutions derived from $BaTiO_3$ and $KNbO_3$). Thus, even though $NaNbO_3$ nominally does not show ion arrangement disorder, it is difficult to generalize the demonstration of relaxor behavior in $NaNbO_3$ to standard FE perovskites

due to *i*) the possibility of relaxor behavior in $NaNbO_3$ being driven by the presence of defects due to the extreme sensitivity of the $NaNbO_3$ structure, and *ii*) due to the fundamental difference in the structural origin of ferroic behavior between $NaNbO_3$ and standard FE perovskites. Therefore, our demonstration of relaxor behavior in high-quality single-crystal PMW films which exhibit long-range cation order does introduce a new paradigm for relaxor behavior for standard perovskite ferroelectric materials which previously were not reported to exhibit relaxor dispersion in systems without cation disorder. We have modified the text in the paper to mention the dispersion previously observed in NBT and $NaNbO_3$ systems and explain the distinction between the relaxor behavior in these systems and in our PMW films to better describe the novelty of our work.

"*While relaxor dispersion in the vicinity of AFE-FE phase transition has been previously observed in $Na_{0.5}Bi_{0.5}TiO_3$ (NBT) and $NaNbO_3$, both of these systems are distinct from the PMW films examined in this study. NBT films show strong cation disorder on the A-site thus relaxor behavior observed in this system is considered to arise from cation disorder, similar to the classic PMN material. While $NaNbO_3$ is nominally ion-disorder-free, it is a highly complex material that is known to be extremely sensitive to a wide variety of factors during synthesis including small deviations from stoichiometry and homogeneity; thus, due to its extreme sensitivity, even low concentrations of defects may affect $NaNbO_3$ strongly enough to give rise to relaxor behavior. Furthermore, the FE and AFE phase in $NaNbO_3$ are due to the off-center displacements of O anions rather than the cation as is the case in PMW and other standard ferroelectrics such as PMN and PZT. Therefore, the relaxor behavior in $NaNbO_3$ can be considered to be a result of the unusual structural properties of $NaNbO_3$. Thus, due to these distinctions between our systems and the previously reported NBT and $NaNbO_3$, our work on high-quality, single-crystal PMW films demonstrates a new paradigm for designing relaxor behavior in standard cation-driven perovskite ferroelectrics through engineered phase competition.*

Raman Spectroscopy:

How is the intermediate orthorhombic phase between 100 and 200 K different from Pnma? Authors report an orthorhombic antiferroelectric phase below T ~ 100 K. Explain how it is different from the high temperature phases (crystal structures). How do you link it with AFE-FE transition?

Response: We appreciate the reviewer's comment. The intermediate orthorhombic phase is still Pnma, as seen in previously reported work. The main difference in this 100K-200K temperature range is the observation of significant narrowing in Raman linewidth with decreasing temperature (*right*), indicative of an increasing length scale of ordering and a coalescing of the structural phase. In previous reports on bulk PMW (A. Kania, *et al.*, *J. Phys.: Condens. Matter*, 8, 4441-4453, 1996; G. Baldinozzi, *et al.*, *J. Phys.: Condens. Matter* 7, 8109-8117, 1995.) changes have been observed in linewidth between Tc and the intermediate phase (in the range of ≈250-315 K) as well as upon the approach to the transition to the lowest temperature AFE phase (≈200-100 K).

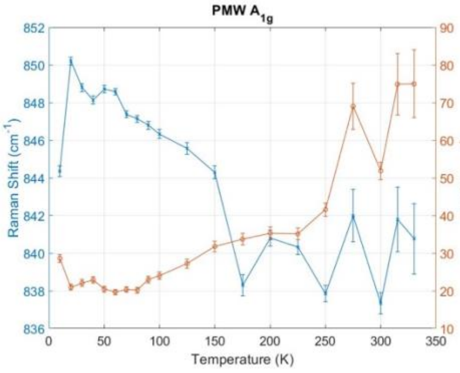

We were unable to use Raman scattering to discern differences between AFE and FE in the lowest temperature phase in our films.

Deconvoluting the peaks in the Raman spectra and plotting their FWHM as a function of temperature may help in fixing the critical temperatures and throw light on various phase transitions in the materials at atomic level.

Response: Deconvolution of the A_{1g} mode (≈840 cm^{-1}), related to the *B*-site ordering stability, supports the claim of long scale phase ordering changes upon lowering the temperature to ≈100 K, all while remaining in the *Pnma* structure. As seen in the previous reports, this mode undergoes changes just after cooling below T_C, stabilizes ≈ 225 K, then noticeably sharpens between ≈200 K - 100 K, stabilizing at ≈ 90K and remaining unchanged until the lowest temperatures.

In conclusion, the presented results do not introduce any novel scientific findings nor do they advance the existing understanding in this area of research. The authors' primary claim—that "these findings demonstrate a new paradigm for designing relaxor material properties through engineered phase competition"—has already been reported in earlier studies where similar phenomena have been observed in pure $NaNbO_3$ and $Na_{1/2}Bi_{1/2}TiO_3$ systems, where the antiferroelectric-to-ferroelectric phase transition is accompanied by diffuse dielectric behaviour exhibiting relaxor-like characteristics.

Response: We thank the reviewer for giving us the opportunity to clarify the significance and novelty of our work. As discussed above, we acknowledge that relaxor-like behavior has been reported to be associated with AFE-to-FE phase transitions in several material systems. However, we would like to emphasize that our study on $PbMg_{0.5}W_{0.5}O_3$ (PMW) differs fundamentally from the previously reported pure $NaNbO_3$ and $Na_{1/2}Bi_{1/2}TiO_3$ (NBT) systems in terms of material system, research motivation, and core findings.

Our work focuses on a lead-based PMW relaxor ferroelectric with full cation ordering, in contrast to NBT for which strong Na/Bi arrangement disorder is present on the A-site cation sublattice and is the most likely origin of the relaxor behavior in NBT. Some ion disorder may also be present in $NaNbO_3$ and give rise to relaxor behavior due to extreme sensitivity of $NaNbO_3$ structure. Additionally, the AFE and FE phases in PMW arise from off-center cation displacements, in contrast to the anion-driven ferro- and antiferroelectric phases in $NaNbO_3$.

Through deliberate materials design and the growth of high-quality epitaxial thin films, we have realized a lead-based system with long-range *B*-site ordering, while minimizing the influence of defects such as oxygen vacancies and disorder. By suppressing extrinsic effects and accessing the intrinsic structural and electrical responses, our findings provide a new, clean experimental perspective for understanding the microscopic origin of dielectric relaxation driven by AFE–FE phase competition. We therefore believe that our work does provide a novel paradigm for designing and engineering relaxor properties through controlled phase competition, and advances the fundamental understanding of relaxor ferroelectrics in this important materials family.

In addition, many of the explanations provided in the manuscript would benefit from further elaboration, additional experiments, and a more detailed analysis of the experimental results. Improved integration of observations from different experimental techniques may help and clarify the scientific context. Accordingly, I do not recommend the manuscript for publication in Nature Communications in its present form.

Response: We sincerely appreciate the reviewer's constructive and insightful comments, which have greatly helped us improve the quality of our manuscript. We believe that all the issues raised by the reviewer have been fully addressed in the revised version. We are confident that the manuscript has been substantially improved and we believe it is now suitable for publication in *Nature Communications*.

References:

1. Mishra, S. K., Mittal, R., Pomjakushin, V. Y., & Chaplot, S. L. (2011). Phase stability and structural temperature dependence in sodium niobate: a high-resolution powder neutron diffraction study. Physical Review B—Condensed Matter and Materials Physics, 83(13), 134105.

2. Lanfredi, S., Lente, M. H., & Eiras, J. A. (2002). Phase transition at low temperature in $NaNbO_3$ ceramic. Applied physics letters, 80(15), 2731-2733.

3. Dorcet, V., Trolliard, G., & Boullay, P. (2008). Reinvestigation of phase transitions in $Na_{0.5}Bi_{0.5}TiO_3$ by TEM. Part I: First order rhombohedral to orthorhombic phase transition. Chemistry of Materials, 20(15), 5061-5073.

4. Isupov, V. A. (2005). Ferroelectric $Na_{0.5}Bi_{0.5}TiO_3$ and $K_{0.5}Bi_{0.5}TiO_3$ perovskites and their solid solutions. Ferroelectrics, 315(1), 123-147.

5. Sakata, K., & Masuda, Y. (1974). Ferroelectric and antiferroelectric properties of $(Na_{0.5}Bi_{0.5})TiO_3$-$SrTiO_3$ solid solution ceramics. Ferroelectrics, 7(1), 347-349.

REVIEWER COMMENTS

Reviewer #1 (Remarks to the Author):

The authors have thoroughly revised the manuscript in response to the reviewers' comments. All previously raised issues have been resolved. I therefore recommend the manuscript for acceptance.

Response: We thank the reviewer.

Reviewer #2 (Remarks to the Author):

The authors respond my comments well, it can be accepted as it is.

Response: We thank the reviewer.

Reviewer #3 (Remarks to the Author):

The authors have made significant effort to revise the manuscript and incorporate the suggested changes, though the manuscript can still be improved with further clarification and better presentation of the results.

1. *The authors state that PbMg0.5W0.5O3 is fully cation-ordered compared to Na0.5Bi0.5TiO3, while both materials still exhibit cation disorder at the B- and A-sites, respectively. Therefore, if the relaxor behaviour in NBT is attributed to cation disorder, a similar interpretation may also apply to PMW.*

Response: We greatly appreciate the reviewer for recognizing our efforts in revising the manuscript and acknowledging our work. We also thank the reviewer for providing valuable and constructive comments. We would like to emphasize that the *B*-site cations in PMW are fully ordered. The ordering diffraction peak in the XRD pattern (Fig. 1f) and the HAADF-STEM image (Fig. 2b) provide macroscopic and microscopic evidence for this cation ordering, respectively. In particular, the HAADF-STEM image clearly reveals the rock-salt-type ordering of the *B*-site sublattice, and no obvious *B*-site cation mixed occupancy is detected in our experimental observations. Therefore, the disordered cation arrangement that has been invoked to explain relaxor behavior in NBT and other lead-containing relaxors is unlikely to be the origin of the relaxor behavior in PMW.

2. *In the deconvolution of the A1g mode (~840 cm-1), the plotted Raman shift shows several anomalies. What do these anomalies correspond to? The FWHM is expected to increase with temperature, but an anomaly is observed around 300 K that requires further explanation.*

Response: An anomaly in deconvolved Raman linewidth (unusual decrease with temperature) can be associated with and attributed to a change in phonon lifetime. We propose that this phenomenon could be caused, *e.g.*, by emergent competition among two phases, which acts as a precursor of structural phase transition. Such phase competition can be accompanied by a divergence in the spatial correlation of the order parameter, leading to the extended phonon coherence length. While an intriguing observation, verifying this hypothesis would require additional validation and investigation using other techniques and theoretical model calculations, which is beyond the scope of the present work.
